# Innovative Multivariable Model Combining MRI Radiomics and Plasma Indexes Predicts Alzheimer’s Disease Conversion: Evidence from a 2-Cohort Longitudinal Study

**DOI:** 10.34133/research.0354

**Published:** 2024-04-16

**Authors:** Xianfeng Yu, Xiaoming Sun, Min Wei, Shuqing Deng, Qi Zhang, Tengfei Guo, Kai Shao, Mingkai Zhang, Jiehui Jiang, Ying Han, for the Alzheimer’s Disease Neuroimaging Initiative

**Affiliations:** ^1^ Department of Neurology, Xuanwu Hospital of Capital Medical University, Beijing 100053, China.; ^2^ Institute of Biomedical Engineering, School of Life Science, Shanghai University, Shanghai 200444, China.; ^3^ Institute of Biomedical Engineering, Shenzhen Bay Laboratory, Shenzhen 518132, China.; ^4^ German Center for Neurodegenerative Diseases (DZNE), 53127 Bonn, Germany.; ^5^ Center of Alzheimer’s Disease, Beijing Institute for Brain Disorders, Beijing 100069, China.; ^6^ National Clinical Research Center for Geriatric Disorders, Beijing 100053, China.

## Abstract

To explore the complementary relationship between magnetic resonance imaging (MRI) radiomic and plasma biomarkers in the early diagnosis and conversion prediction of Alzheimer’s disease (AD), our study aims to develop an innovative multivariable prediction model that integrates those two for predicting conversion results in AD. This longitudinal multicentric cohort study included 2 independent cohorts: the Sino Longitudinal Study on Cognitive Decline (SILCODE) project and the Alzheimer Disease Neuroimaging Initiative (ADNI). We collected comprehensive assessments, MRI, plasma samples, and amyloid positron emission tomography data. A multivariable logistic regression analysis was applied to combine plasma and MRI radiomics biomarkers and generate a new composite indicator. The optimal model’s performance and generalizability were assessed across populations in 2 cross-racial cohorts. A total of 897 subjects were included, including 635 from the SILCODE cohort (mean [SD] age, 64.93 [6.78] years; 343 [63%] female) and 262 from the ADNI cohort (mean [SD] age, 73.96 [7.06] years; 140 [53%] female). The area under the receiver operating characteristic curve of the optimal model was 0.9414 and 0.8979 in the training and validation dataset, respectively. A calibration analysis displayed excellent consistency between the prognosis and actual observation. The findings of the present study provide a valuable diagnostic tool for identifying at-risk individuals for AD and highlight the pivotal role of the radiomic biomarker. Importantly, built upon data-driven analyses commonly seen in previous radiomics studies, our research delves into AD pathology to further elucidate the underlying reasons behind the robust predictive performance of the MRI radiomic predictor.

## Introduction

Alzheimer’s disease (AD) is a progressive neurodegenerative disease that impacts tens of millions of people worldwide and places important medical burdens on the public health system [1]. Due to the absence of an effective strategy to delay or cease the progression [2,3], the irreversibility nature of AD attracts significant attention to early diagnosis and conversion outcome prediction during the preclinical stage, commencing 15 to 20 years prior to the onset of clinical symptoms [2,4]. Since the publication of the 2018NIA-AA research framework [2], disease-targeted therapies have received regulatory approval, and plasma biomarkers with great diagnostic performance have been developed. As a result, the research framework that was originally designed for research purposes now needs to be revised to guide both research and administering clinical treatment.

In the 2018 Alzheimer’s Association International Conference (AAIC), a research framework for AD including 3 biomarker groupings was presented: aggregated Aβ (A), aggregated tau (T), and neurodegeneration or neuronal injury (N) [2]. The research framework was abbreviated as ATX(N), where X denoted possibilities to incorporate new biomarkers [2]. Excitingly, in the 2023 AAIC, a new biomarker categorification was introduced for staging and AD prognosis: biomarkers of inflammatory/immune processes (I), currently only reflected by body fluid, e.g., plasma or cerebrospinal fluid (CSF) glial fibrillary acidic protein (GFAP) [5]. Fluid markers and imaging markers are accurately measured by CSF-based biomarkers and positron emission tomography (PET), respectively. However, these 2 methods are incompatible with community health centers [6] due to their high costs and safety concerns [7,8]. As an alternative, plasma biomarkers and magnetic resonance imaging (MRI) are more clinically feasible and accessible to the public. Plasma biomarkers for Aβ42/40, p-tau 181, neurofilament light chain (NfL), and GFAP are suggested to be informative in addition to CSF [5], and specific MRI radiomics features may offer thorough and sensitive information about various brain regions, revealing AD pathological mechanisms and facilitating early diagnosis [9]. While both plasma biomarkers and MRI markers are informative in reflecting different aspects of AD pathology, relying solely on plasma or MRI biomarkers when other techniques are not available may raise concerns: the limited exchange of proteins between plasma and brain extracellular fluid [10] makes it challenging to precisely track longitudinal changes of AD pathology, and neurodegeneration reflected by MRI is not specific to AD compared to the core biomarker (A and T) [2,5].

Alternatively, a combination of plasma and MRI biomarkers holds great potential for improving the accuracy of predicting AD conversion outcomes, given their complementary nature in capturing different aspects of the disease progression. Therefore, this paper proposes a novel multivariable prediction model combining plasma and MRI radiomics biomarkers. By exploring the combination of these 2 techniques, we hope to provide a more accurate and efficient diagnostic tool for AD, ultimately leading to better patient outcomes. The present study aims to (a) follow the ATN framework of plasma in combination with MRI to trace longitudinal changes from cognitively unimpaired (CU) to cognitive impairment (CI); (b) validate the clinical efficacy of the composite indicator by using longitudinal data and explore the underlying pathological mechanism through correlation analysis; and (c) build upon previous radiomics studies and delve beyond data-driven analyses to elucidate the underlying mechanisms behind Rad’s robust predictive performance.

## Results

### Participants

**Fig. 1.**
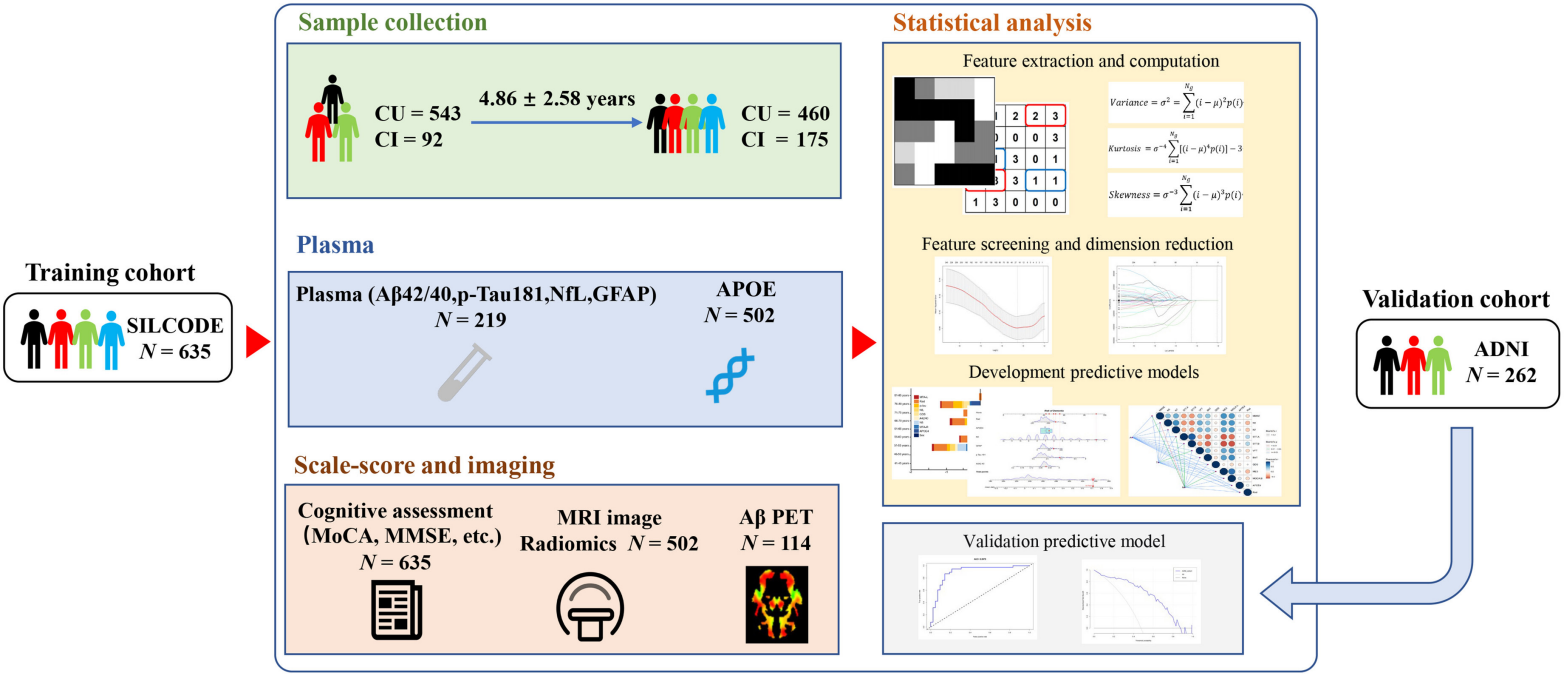
Flow diagram of the primary study. CU, cognitively unimpaired; CI, cognitive impairment; APOE, apolipoprotein E; NfL, neurofilament light chain; GFAP, glial fibrillary acidic protein; MoCA-B, Montreal Cognitive Assessment Scale; MMSE, Mini-Mental State Examination test.

Demographic and clinical characteristics at baseline and during follow-ups (4.86 ± 2.58 years) of 635 participants can be found in Table. Because 92 participants were in the CI at baseline, they were excluded from the subsequent prediction model (Fig. 1). Between CU and CI subgroups, differences in mean age and education time were found to be important at baseline (*P* < 0.001), but during follow-ups, only the difference in education time remained marked and a significant gender difference emerged (*P* < 0.05). As expected, at any time point, substantial differences were found in plasma biomarkers (Aβ42/40, P-tau 181, NfL, and GFAP) between CU and CI groups (*P* < 0.05). In neuropsychological evaluations (Mini-Mental State Examination [MMSE], Montreal Cognitive Assessment Scale [MoCA], Auditory Verbal Learning Test [AVLT], Shape Trail Test [STT], memory and executive screening [MES], Verbal Fluency Test [VFT], Boston Naming Test [BNT], and Clinical Dementia Rating scale [CDR] scores), participants in the CI group got significantly lower scores than those in the CU group (*P* < 0.05). However, no obvious difference was found between the 2 groups in some other psychological evaluations (Hamilton Anxiety Scale [HAMA], GDS, etc.) (*P* > 0.05). Within the CI group, the carrier rate of the APOE ε4 allele in the participants significantly increased from baseline to follow-up. Additionally, the SUVR values between the 2 groups show great differences (*P* < 0.001) in both baseline and follow-up.

**Table.** Demographic information and clinical characteristics at baseline and follow-up time

	Baseline			Follow-up		
	CU	CI	*P* value	CU	CI	*P* value
Sex (female, *n*, %)	343, 63.17%	53, 57.72%	0.251	275, 59.71%	91, 51.79%	<0.001
Age (years)	64.93 ± 6.78	70.47 ± 9.25	<0.001	66.62 ± 6.63	68.35 ± 9.64	0.188
Education (years)	11.89 ± 3.78	10.01 ± 4.98	<0.001	11.97 ± 3.77	10.75 ± 3.89	0.032
Plasma Aβ42/40	0.06 ± 0.02	0.05 ± 0.01	0.021	0.06 ± 0.01	0.05 ± 0.01	0.035
Plasma P-tau 181 (pg/ml)	1.85 ± 0.75	3.07 ± 1.31	<0.001	2.23 ± 1.13	4.92 ± 0.71	0.003
Plasma NfL (pg/ml)	14.81 ± 6.76	21.14 ± 10.37	0.002	16.70 ± 8.20	37.32 ± 14.90	<0.001
Plasma GFAP (pg/ml)	115.07 ± 55.81	210.55 ± 121.85	<0.001	129.58 ± 61.96	270.80 ± 70.69	<0.001
HAMD	4.05 ± 4.19	6.16 ± 6.98	0.017	3.27 ± 3.63	4.00 ± 2.60	0.551
HAMA	4.45 ± 3.97	5.84 ± 5.54	0.086	3.81 ± 3.45	4.22 ± 2.95	0.727
MMSE	28.41 ± 1.75	22.48 ± 4.72	<0.001	28.49+1.66	22.96 ± 5.18	<0.001
AVLT-N5	6.96 ± 2.06	2.82 ± 2.48	<0.001	7.66 ± 2.24	1.67 ± 2.19	<0.001
AVLT-N7	22.20 ± 1.90	18.05 ± 3.65	<0.001	22.41 ± 1.67	17.75 ± 3.31	<0.001
STT-A	61.30 ± 19.01	100.96 ± 54.21	<0.001	59.01 ± 20.44	102.89 ± 44.47	<0.001
STT-B	138.55 ± 39.85	236.42 ± 142.65	<0.001	142.79 ± 45.64	182.44 ± 46.95	<0.001
VFT	18.91 ± 4.62	13.91 ± 4.68	<0.01	19.01 ± 4.38	14.80 ± 4.26	0.012
BNT	24.79 ± 3.12	19.41 ± 4.92	<0.001	25.39 ± 2.87	21.50 ± 2.17	<0.001
GDS	2.50 ± 2.46	3.35 ± 3.06	0.081	2.34 ± 2.10	3.30 ± 2.91	0.168
MES	90.26 ± 7.02	69.35 ± 15.26	<0.001	91.05 ± 7.01	74.80 ± 22.13	<0.001
PSQI	5.04 ± 3.46	3.89 ± 2.68	0.05	4.81 ± 3.20	6.90 ± 4.65	0.051
RBDSQ	1.35 ± 1.86	0.85 ± 1.54	0.132	1.11 ± 1.50	2.20 ± 2.44	0.033
ESS	7.34 ± 4.80	4.72 ± 4.99	0.018	6.76 ± 4.65	7.00 ± 4.42	0.871
MoCA-B	25.57 ± 2.55	18.87 ± 4.79	<0.001	25.78 ± 2.61	20.17 ± 4.30	<0.001
CDR	0.03 ± 0.13	0.72 ± 0.47	<0.001	0.03 ± 0.11	0.75 ± 0.57	<0.001
APOEε4 (carrier, *n*, %)	78, 22.10%	64, 42.95%	<0.001	61, 21.94%	104, 46.43%	<0.001
SUVR	0.97 ± 0.97	1.32 ± 0.29	<0.001	0.99 ± 0.10	1.36 ± 0.27	<0.001

HAMD, Hamilton Depression Scale; HAMA, Hamilton Anxiety Scale; MMSE, Mini-Mental State Examination test; AVLT-N5: Auditory Verbal Learning Test-Huashan version long-delayed free recall (20 min); AVLT-N7: AVLT-Huashan version long-delayed recognition (20 min); STT-A: Shape Trail Test A; STT-B: Shape Trail Test B; VFT, Verbal Fluency Test (animal); BNT, Boston Naming Test; GDS, Geriatric Depression Scale; MES, memory and executive screening; PSQI, Pittsburgh Sleep Quality Index; RBDSQ, Rapid-eye-movement Sleep Behavior Disorder Screening Questionnaire; ESS, Epworth Sleepiness Scale, MoCA-B, Montreal Cognitive Assessment Scale; CDR, Clinical Dementia Rating scale; Rad, Radiomics score.

### MRI radiomics biomarkers extraction

**Fig. 2.**
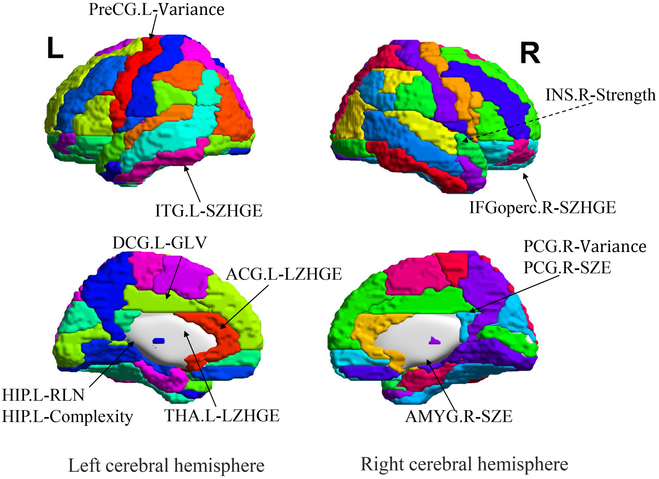
The 12 imaging genomics features included in the LASSO analysis and their spatial locations. PreCG.L-Variance, Precentral gyrus-Variance; IFGoperc.R-SZHGE, Inferior frontal gyrus, opercular part-Small Zone High Gray-level Emphasis; INS.R-Strength, Insula-Strength; ACG.L-LZHGE, Anterior cingulate and paracingulate gyri-Large Zone High Gray-level Emphasis; DCG.L-GLV, Median cingulate and paracingulate gyri-Gray-level Variance; PCG.R-Variance, Posterior cingulate gyrus-Variance; PCG.R-SZE, Posterior cingulate gyrus-Small Zone Emphasis; HIP.L-RLN, Hippocampus-Run-Length Non-uniformity; HIP.L-Complexity, Hippocampus-Complexity; AMYG.R-SZE, Amygdala-Small Zone Emphasis; THA.L-LZHGE, Thalamus-Large Zone High Gray-level Emphasis; ITG.L-SZHGE, Inferior temporal gyrus-Small Zone High Gray-level Emphasis.

In the radiomics features, among 3,870 features in the main cohort (Figs. S2A and B), those with non-zero coefficients were included in the least absolute shrinkage and selection operator (LASSO) regression model. Therefore, 12 imaging genomics features were included and their spatial locations are detailed in Fig. 2. All 12 features, in accordance with the Rad score calculation formula as well as the scores for all patients, are provided in the Supplementary Materials (Fig. S3). To decipher the selection process, we conducted correlation analyses between each genomics feature and plasma biomarkers. The results showed that various features are significantly correlated with plasma biomarkers, providing strong pathological justification for why those 12 genomics features could be potential components for the Rad biomarker (Fig. S4). For example, GFAP was found to be significantly correlated with the following 4 genomics features: HIP.L-RLN (Hippocampus-Run-Length Non-uniformity), HIP.L-Complexity (Hippocampus-Complexity), PCG.R-SZE (Posterior cingulate gyrus-Small Zone Emphasis), and ITG.L-SZHGE (Inferior temporal gyrus-Small Zone High Gray-level Emphasis). In addition, PCG.R-SZE and ITG.L-SZHGE were found to be significantly correlated to plasma p-tau181 and Aβ42/Aβ40, respectively.

We calculated the Rad scores for each of the 262 subjects in the imaging data to verify our Rad biomarker using data from the Alzheimer Disease Neuroimaging Initiative (ADNI), and we found that the area under the receiver operating characteristic (ROC) curve (AUC) for this dataset was 0.6950, which was lower than the training dataset’s AUC of 0.7678 (Fig. S5). In addition, a Delong test suggested that no obvious difference was found between the 2 datasets. The calibration plots also demonstrated effective model calibration, as evidenced by the outstanding agreement between the anticipated likelihood of prognosis and actual observation (Fig. S6).

### Plasma and MRI radiomics biomarkers combined prediction models

**Fig. 3.**
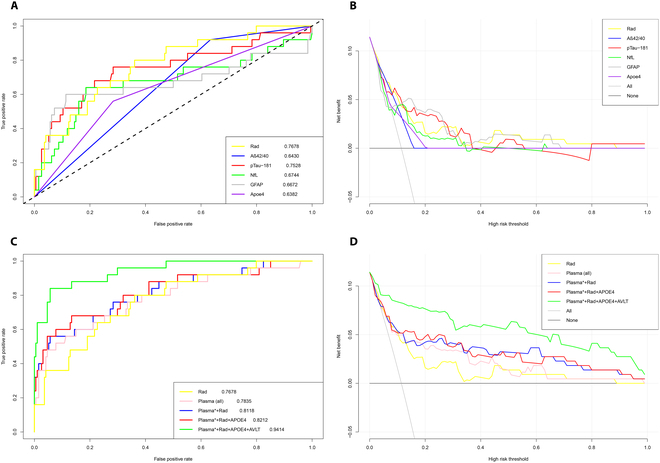
A and C show the ROC and the decision curve analysis of using each biomarker; B and D show the ROC and the decision curve analysis of using various models combining plasma and radiomic indicators. In B and D, the *Y*-axis measures net benefits. The gray line represents the scenario in which all patients would transform to CI, and the thin black line represents the scenario in which no patient transforms to CI. The net benefit was calculated by subtracting the proportion of all false-positive patients from the proportion of true positives, weighted by the loss brought by no treatment to CI and unnecessary treatment.

Rad alone had an accuracy rate of 0.7678 in predicting the conversion outcome of CU, while Aβ42/40 alone had a rate of 0.6430, NfL had a rate of 0.6744, GFAP had a rate of 0.6672, APOE ε4 had a rate of 0.6382, and p-tau181 had a rate of 0.7528 (Fig. 3A). In addition, the accuracy of AVLT-N5 and -N7 in predicting the outcome of CU was 0.7859 and 0.7091, respectively. The accuracy rate of the combined ROC of plasma (all) was 0.7835, which was not significantly different from the Rad model (Delong test, *P* > 0.05). The AUC was 0.8118, 0.8212, and 0.9414 (sensitivity: 0.8, specificity: 0.98), respectively, after successively integrating Plasma (Aβ42/40, p-tau181, and GFAP) (Fig. S7), APOE ε4, and AVLT (Fig. 3C). Thus, after comparing the Akaike information criterion (AIC) and Bayesian Information Criteria (BIC) [11], Aβ42/40, p-tau181, GFAP, APOE ε4, AVLT-N5, and Rad were selected as the optimal model. We used data from ADNI to validate the predictive power of the optimal model. Unfortunately, due to the lack of GFAP data, only Aβ42/40, p-tau181, APOE ε4, AVLT-N5, and Rad data were used for the validation process. In the validation cohort, our optimal model’s AUC (without GFAP) for the external validation cohort was 0.8979 (sensitivity: 0.74, specificity: 0.84) (Fig. S8), which is a respectable indicator of its predictive power.

**Fig. 4.**
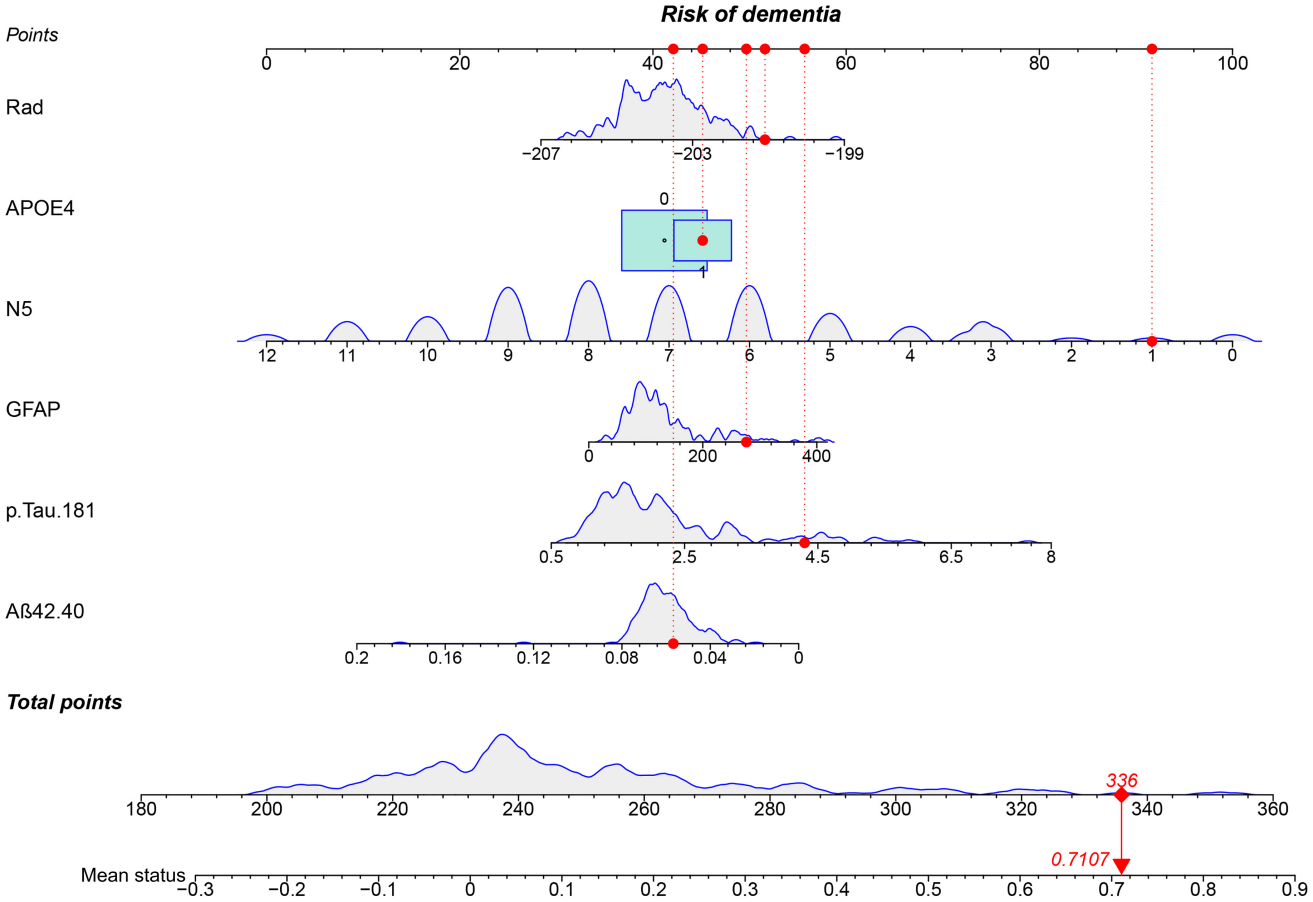
The nomogram shows the calculation of the final probability of the adverse outcome of CU converting to CI in the future. First, a total point was determined based on individual predictor points calculated using the nomogram: a vertical line was drawn from each predictor’s axis to the “point” axis to obtain an individual predictor point, all of which were then summed up to form the aforementioned total point. Then, another vertical line was drawn from the “total point” axis to the “risk” axis to finally get the probability of conversion from CU to CI.

The nomogram in this study (Fig. 4) shows the calculation of the final probability of the adverse outcome of CU converting to CI in the future. First, a vertical line was extended from each predictor’s axis to the “points” axis to receive an individual predictor point, all of which were then summed up to form the total points. Then, another vertical line was drawn from the “total points” axis to the “risk” axis to finally get the probability of conversion from CU to CI. The total points for the majority of the participants in this research ranged from 180 to 360. For example, a randomly selected patient has a Rad score of −201.09, carrying APOE ε4, an AVLT-N5 of 1, a GFAP of 276.46, a P-tau181 of 4.30, and an Aβ42/40 of 0.06; hence, the points for each predictor would be 52.3, 45, 92, 49.6, 55.6, and 41.5, respectively. Thus, we could obtain a total point of 336, and for this particular patient, the predicted probability of converting from CU to CI in the future is approximately 71.07%.

### Clinical performance of the model

Figure 3B and D show the decision curve analyses of each biomarker and various radiomics models [12]. Compared to treating all patients or none, we observed an increase in net benefits when using the radiomics nomogram to predict the conversion from CU to CI with a threshold probability over 8% (Fig. 3D). For instance, if a patient has an individual threshold probability of 60% (meaning that the patient has a greater than 60% chance of converting from CU to CI), the net benefit of using the optimal radiomics nomogram to decide whether to undergo treatment is 0.03, indicating additional benefits compared to treating all patients or none of them. We also conducted decision curve analysis on the optimal model using data from the ADNI cohort and found that net benefits rise as the threshold probability exceeds 10% (Fig. S9).

### Correlation and mediating analysis

**Fig. 5.**
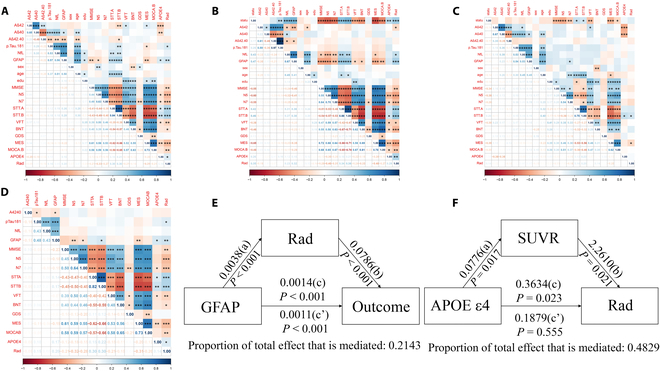
A series of correlation analyses presented as heatmaps in A to D. A presents the correlation heatmaps for all patients; B and C present the correlation heatmaps for patients grouped by high and low Rad scores; D shows the correlations after correcting for sex, age, and education. The correlation coefficient *r* is given in the heatmaps and the asterisks (*, **, and ***) indicate statistical significance at *P* < 0.05, *P* < 0.01, and *P* < 0.001, respectively. E and F present the mediator analysis associated with Rad.

We carried out a series of correlation analyses presented as heatmaps in Fig. 5A to D. In Fig. 5A, in addition to the substantial correlation between age and all plasma biomarkers, we observed that both APOE ε4 and Rad were correlated with GFAP and that Rad was also correlated with MMSE, STT, AVLT, MoCA, and other cognitive and behavioral measures. Figure 5B and C present the correlation heatmaps for patients categorized by high and low Rad scores, indicating that the Rad+ group had significantly stronger correlations than the Rad− group. In the Rad+ group, Rad was strongly correlated with GFAP (*P* < 0.001) and correlated with NfL (*P* < 0.05). Figure 5D shows the correlations after accounting for sex, age, and education.

Rad was found to mediate the effects of GFAP on the conversion outcome of CU, accounting for 21.43% of the overall impact in the mediating effect analysis (Fig. 5E). In addition, Rad also mediates the effects of GFAP on the results of MMSE, contributing 18.60% to the overall impact (Fig. S10). Rad was also found to be able to mediate the effects of NfL on the conversion outcome of CU, accounting for 21.35% of the overall impact (Fig. S11). Interestingly, the effect of APOE ε4 on Rad was completely mediated by SUVR, accounting for 48.29% of the total effect (Fig. 5F).

## Discussion

Due to AD’s high heterogeneity and irreversible nature, it is essential to accurately predict the future conversion in at-risk populations. The ATNI system, as a biomarker system for staging and prognosis, offers substantial help to customize the AD risk profile by sorting pathologic changes into 4 categories [13]. In light of that, our study presents an optimal model that incorporates Rad, APOE ε4, Aβ42/40, P-tau18, GFAP, and AVLT-N5. Our model retains an AUC of 0.9414 in predicting the conversion from CU to CI, increasing the accuracy compared to earlier research.

A highlight of the optimal model is the construction of the Rad biomarker, which had the highest AUC of 0.7678. In radiomics, 3,870 features of the whole brain were combined to create Rad factors, which indicate changes in high-dimensional aspects of the whole brain. The final Rad biomarker included features that match the appropriate brain areas potentially subject to AD pathology in earlier study findings. To be more specific, studies have shown that the morphological changes in mild cognitive impairment (MCI) brain amyloidosis include distinctive spatial patterns that are highly susceptible to AD pathology, such as the central pre-gyrus [14], the entorhinal cortex [15], the right inferior frontal gyrus [16], the insula [17], the posterior cingulate gyrus [18,19], the temporoparietal cortex [20], the hippocampus [21–23], and the thalamus [24]. Additionally, in accordance with a recent clinical follow-up study identifying atrophy in subregions of the amygdala as a potential marker for future progression to CI [25], a subregion of the amygdala was included in our Rad marker, emphasizing the involvement of the amygdala in the early development of AD [26,27] (Fig. 2). All the above discussion provided additional explanations as to why Rad carries the highest AUC. Excitingly, the predictive ability was raised to 0.9414 by incorporating the Rad biomarker and plasma markers indicating A (Aβ42/40), T (P-tau181), I (GFAP), APOE ε4, and cognitive function status (AVLT-N5) in the optimal model. For future applications of the optimal model in clinical use, we have developed a personalized nomogram for predicting conversion from CU to CI. The DCA demonstrated that our nomogram for predicting survival rates was more useful and practical than the traditional ATN [2] staging system. With advancements in neuroscience and brain science, machine learning and various algorithms are continuously evolving [28]. Overall, our nomogram may be a valuable tool for prognosis prediction in the Chinese community.

**Fig. 6.**
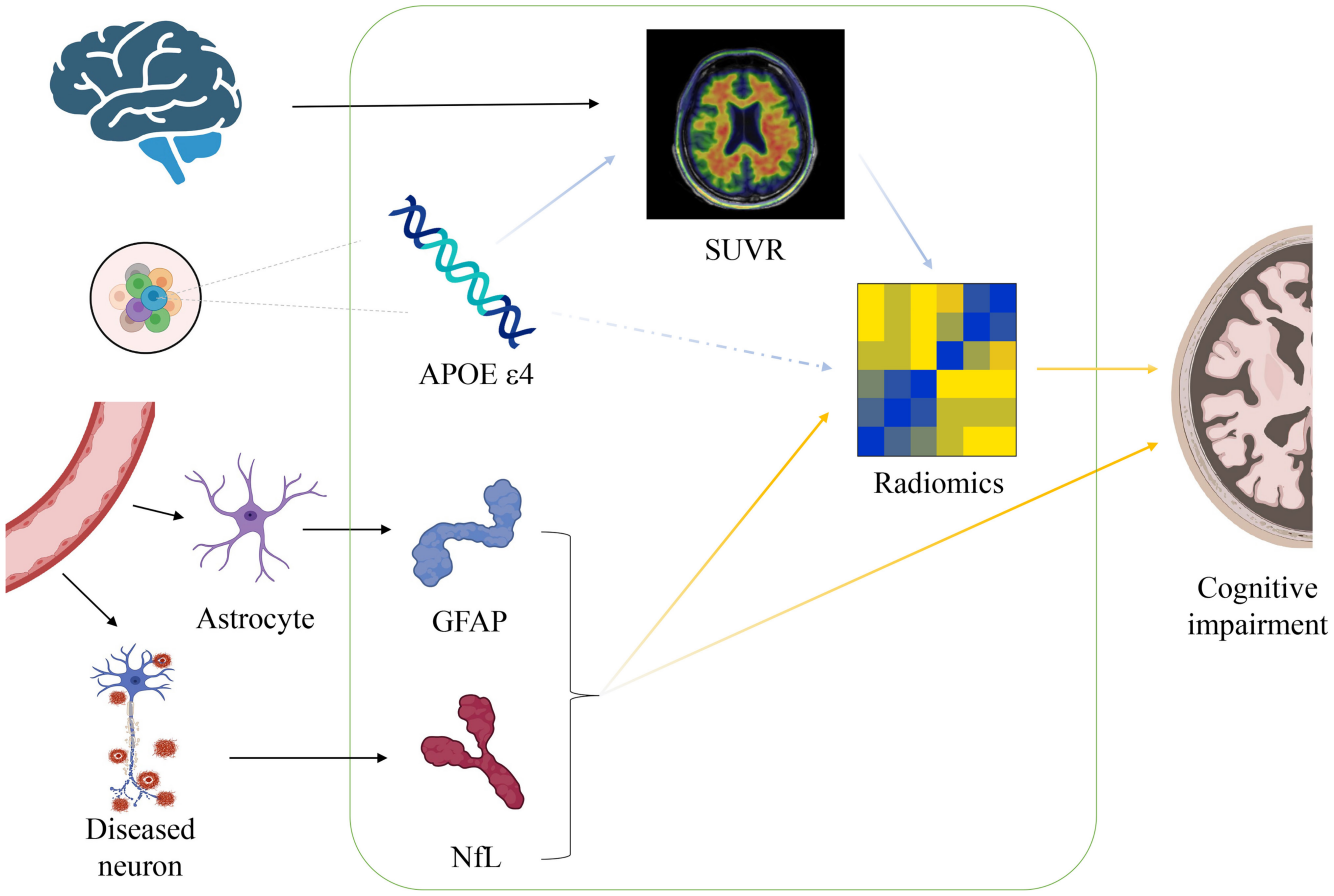
Possible pathways for the conversion from CU to CI.

Numerous factors were found to be mediating the conversion after a mediator analysis was conducted (Fig. 6). One finding that stands out is that the effect of APOE ε4 on Rad was entirely mediated by the deposition of Aβ in the brain, which is in line with research done by Dincer et al. [29] and Salami et al. [30], suggesting that the APOE genotype may have an influence on longitudinal changes in radiomics such as MRI [31,32]. In addition, in accordance with past studies [33–35], plasma GFAP and NfL were found to have an impact on conversion outcomes both directly and indirectly via Rad. In particular, Rad was strongly correlated to outcome in individuals with positive plasma Aβ (Fig. 5B), highlighting the significance of integrating AD-related imaging and plasma markers to comprehend the conversion process from CU to CI. In addition, Rad served as a mediator between plasma Aβ profile effects and conversion outcome, suggesting that the effects pathway may involve multiple brain regions, multiple dimensions, and even global. Moreover, a highlight of the present study is that we explored changes in Rad existing in high-dimensional space and its impact on low-dimensional image data. Given these factors, our exploration of the potential conversion process from CU to CI in preclinical AD outlined in the present study might aid future research and clinical practice diagnosis.

Additionally, we grouped individuals into high and low Rad score before using Kaplan–Meier curves to examine differences in time points at which conversion from CU to CI occurs between the 2 groups. Results showed that conversion from CU to CI occurred significantly earlier in the high Rad score group than in the low Rad score group (*P* = 0.006, Fig. S12), indicating that even in hospitals in rural or remote areas, where PET, CSF, and plasma tests cannot be performed, using only MRI might be a possible way to identify at-risk individuals. Further research is encouraged to elucidate complex processes underlying the relationship between the MRI data and conversion outcome.

The NIA-AA draft criteria for AD demonstrated that imaging and fluid biomarkers are not interchangeable within the same category [5]. Ideally, a complete biomarker profile should include both fluid and imaging biomarkers as each captures different aspects of the AD pathology. However, each biomarker comes with different sensitivities and specificities for various use cases, and building a diagnostic tool to identify at-risk individuals on a large scale must consider realistic constraints. In the category A and T, compared to imaging markers, fluid markers are considered more sensitive to early changes in AD, which aligns with the aim of the present study to identify at-risk individuals and predict the conversion from CU to CI [5]. In category N, our final model only included the imaging biomarker (Rad) but not the fluid biomarker (NfL). We ran AIC and BIC analysis on the optimal model and a more complicated model (the optimal model + NfL). The optimal model (AIC: 81.99, BIC: 105.71) was considered to be the most optimal compared to the complicated model (AIC: 83.66, BIC: 110.77), as it prevented the model from being overly complicated or overfitting.

For our current work, several limitations need to be addressed. First, 8 patients converted from CU to CI in our most recent large-scale follow-up; thus, some data in the CU group may pertain to people who are in the pre-conversion stage to CI. In the future, we will carry out continuing follow-ups to obtain more stable results. In addition, the length of our study’s follow-up (4.86 ± 2.58 years) may limit the generalizability of our results because the development of AD can persist for up to 10 years. However, given that most AD imaging studies have comparable follow-up times and that Rad score can identify significant pathological changes, we believe that the impact of this relatively short follow-up period may not be particularly important. Third, we were unable to collect sufficient GFAP data from ADNI; thus, this indicator was left out of the validation process. In addition, a recent longitudinal clinical follow-up study showed that a significant proportion of ADNI subjects classified as healthy controls showed significant signs of amygdala atrophy at baseline [25]. Similarly, a subregion of the amygdala is included in our Rad marker, providing additional evidence for the potential association between early changes in the amygdala and future cognitive changes. As a result, future research is encouraged to further examine the association between the structural integrity of the amygdala and cognitive decline. Finally, the Simoa method for measuring ptau181 began in 2020; thus, plasma samples were available for only 219 patients, and the accuracy of long-term frozen plasma measurements has yet to be validated [36]. However, given the AUC (only Aβ42/40, p-tau181, APOE ε4, AVLT-N5, and Rad included) values, we are confident in the current model’s propensity to accurately predict conversion outcomes.

In conclusion, this study presents an optimal model for predicting the conversion from CU to CI, highlighting the importance of the Rad biomarker and its potential as a valuable tool to identify individuals at risk of developing dementia. Furthermore, the plasma biomarkers GFAP and NfL may also contribute to the eventual onset of dementia by affecting Rad during this longitudinal process. In the future, with the availability of effective AD treatments, our model can aid in identifying individuals who would benefit the most from either primary or secondary prevention. Overall, all the findings offer insight into the pathophysiology of AD and explore the potential for personalized, targeted interventions.

## Methods

### Study population

All participants finished written informed consent and authorized the publication of their clinical details. Approval for the study procedures was granted by relevant ethics committees. Neuropsychological scales, imaging data, and plasma tests (Simoa in some) were assessed at enrollment and follow-up.

### Sino Longitudinal Study on Cognitive Decline project

The present study is part of the Sino Longitudinal Study on Cognitive Decline (SILCODE) project, an ongoing registered multicenter AD research project on the Han Chinese community population in mainland China [37]. Specific inclusion/exclusion criteria can be discovered at https://www.clinicaltrials.gov/ct2/show/study/NCT03370744. From a total of 635 patients, clinical information, neuropsychological assessments, blood samples, and imaging data were collected between January 2010 and December 2022 (Fig. 1).

Subcategories of participants are described as follows: Diagnosing Normal Control (NC) was based on excluding individuals with MCI [13] and dementia [38]. The CU group included individuals who met the NC criteria, and the CI group included those with MCI or dementia. Detailed information on each scale and eligibility criteria can be acquired from the protocol that has been published [37] and studies conducted earlier by us [39,40].

### Alzheimer Disease Neuroimaging Initiative

As a multicentered longitudinal database, ADNI aims at developing clinical, radiomics, and biological indicators, which is suitable for early diagnosis and measuring the progression during the early stages of AD [41]. The dataset we used for validation included CU individuals (262 in total), 168 of whom transformed into CI eventually (adni.loni.usc.edu). The inclusion and exclusion criteria of individuals from the ADNI database were as follows: (a) all individuals who were diagnosed CU at the baseline visit and were followed up for at least 3 years and the CI patients who had converted to MCI or dementia within the follow-up interval were included; (b) individuals with MRI, plasma samples, and amyloid PET data were likewise included; and (c) individuals with a bidirectional change in diagnosis (CU to CI, and back to CU) within the follow-up period were excluded. (d) All individuals who underwent visual assessment by an experienced imaging staff and individuals with significant atrophy on baseline MR images, including amygdala and hippocampus, were excluded. For more updates on ADNI, please refer to www.adni-info.org. The enrollment process of the ADNI data in this study is shown in Fig. S1.

### Neuropsychological assessment

In both cohorts, the cognitive performance of each participant was assessed annually by several neuropsychological assessments, and longitudinal data from both baseline and later follow-ups were included in the analysis. In SILCODE, assessments were designed to cover the main common cognitive domains including memory, language, and executive function. Included measurements were as follows: MMSE [42]; AVLT-N5: AVLT-Huashan version long-delayed free recall (5 min); AVLT-N7: AVLT-Huashan version long-delayed recognition (20 min) [43]; STT-A: Shape Trail Test A; STT-B: Shape Trail Test B [44]; GDS, Geriatric Depression Scale [45]; MES [46]; MoCA-B [47]; CDR [48], etc. Among all, MMSE and MoCA scores were utilized to evaluate overall cognition, and all the scores were *z*-normalized during analysis to eliminate measurement bias. The validation method in ADNI made use of existing longitudinal data for the same metrics.

### Neuroimaging data acquisition and processing

In the context of SILCODE, neuroimaging data acquisition including [18F] florbetapir (AV-45) PET and MRI scans were conducted on a simultaneous 3.0T TOF PET/MR scanner (SIGNA PET/MR, GE Healthcare, Milwaukee, WI, USA) at Xuanwu Hospital of Capital Medical University, Beijing. Participants received an intravenous injection of 7 to 10 mCi [18F] florbetapir radiotracer, followed by a 40-min rest period before undergoing a 20-min static PET scan. The PET data were collected using a time-of-flight ordered subset expectation maximization (TOF-OSEM) algorithm with specific parameters: 8 iterations, 32 subsets matrix = 192 × 192, the field of view (FOV) = 350 × 350 mm^2^, and half-width height = 3. In MRI scans, the parameters for T1-weighted 3D brain structural images were as follows: SPGR sequence, FOV = 256 × 256 mm^2^, matrix = 256 × 256, slice thickness = 1 mm, gap = 0, slice number = 192, repetition time (TR) = 6.9 ms, echo time (TE) = 2.98 ms, inversion time (TI) = 450 ms, flip angle = 12°, and voxel size = 1 × 1 × 1 mm^3^. The MRI images were processed using SPM12 (http://www.fil.ion.ucl.ac.uk/spm/software/spm12). In the pre-processing, (a) the quality of all images was assessed by 2 experienced radiologic technologists, and the considered key metrics included the signal-to-noise ratio (SNR), spatial resolution, and scan time; (b) the DICOM (Digital Imaging and Communications in Medicine) file was converted into a NIfTI (Neuroimaging Informatics Technology Initial) file using the y_Call_dcm2nii function in the DPABI V6.1 toolkit; (c) the T1 image was divided into gray matter, white matter, cerebrospinal fluid tissue probability map, skull, and other tissues (corresponding to c1 to c5) using SPM12; (d) the (c1) space of the segmented cortical images was spatially normalized to the Montreal Neurological Institute (MNI) space, and the voxel size was 2 mm × 2 mm × 2 mm; and (e) the 8 mm × 8 mm × 8 mm isotropic Gaussian smoothing kernel was used for smoothing. To evaluate possible pathways of imagological influence, we used reference regions of the entire cortex to generate a voxel 18F-AV-45 standardized uptake value ratio (SUVR) image: the entire cerebellum, WM (white matter) based on the MNI map, and topic-specific WM. The SUVR of the cortex obtains the count ratio by averaging the voxel SUVR images in the particular target area.

### Model construction

The model was constructed through 3 steps: (a) plasma biomarker extraction; (b) MRI radiomics biomarker extraction; and (c) the optimal model construction that combined the 2 components using the machine learning technique. The training dataset used in the model constructing process is from our SILCODE cohort, and the validation dataset was from ADNI (Fig. 1).

### Plasma biomarker extraction

In SILCODE, P-tau181 concentration was measured by the Single Molecule array (Simoa) p-tau181 Advantage Kit, while Aβ40, Aβ42, NfL, and GFAP concentrations were measured by the Simoa Human Neurology 4-Plex E (N4PE) assay (Quanterix). All measurements for the 5 analytes exceeded the detection limit, with an intra-assay variation coefficient of less than 10%. The data were then matched to phenotype information.

In the ADNI cohort, plasma Aβ42/40 was detected by a high-precision liquid chromatography–tandem mass spectrometry (LC–MS/MS) [49], and plasma p-tau181 was analyzed by the validated ultrasensitive Simoa technique at the Clinical Neurochemistry Laboratory, University of Gothenburg, Sweden [36]. For p-tau181, the lower limit of quantification was 1.0 pg/ml.

### MRI radiomics biomarker extraction

MRI radiomics features were extracted from the processed neuroimaging data with the tools developed by Vallières et al. [50] (https://github.com/mvallieres/radiomics). Based on the brain atlas Automated Anatomical Labeling [51], 90 cortical regions (Nos. 1 to 90) were used as regions of interest, and 43 features (numbered in the order of 1 to 43 in the text) were extracted in each brain region, thus making a total of 43 × 90 = 3,870 features per subject (Supplementary Materials).

To reduce the dimension of features and to select potential predictive factors, the LASSO-based proportional hazards model (LASSO-COX), a model commonly used to evaluate the predictive ability of selected features and to determine the optimal subset of features [52], was constructed. To fully explore potential predictive factors, the leave-one-out cross-validation method was utilized, seeking the optimal solution for the regularization coefficient (λ-value) based on radiomic features within the LASSO-COX model, and thus obtaining the corresponding optimal radiomic features subset.

Finally, the Rad score (a radiomics scoring system that consolidates multiple radiomic features into a single comprehensive index to reflect the biological information in medical imaging) was calculated for each participant via a linear combination of selected features weighted by the coefficients, therefore concluding the image radiomics biomarker.

### Plasma and MRI radiomics biomarkers combined prediction model

In this study, multivariable logistic regression analysis was performed to integrate plasma and radiomics biomarkers using clinical candidate predictors including Aβ42/40, P-tau181, GFAP, AVLT-N5, APOE4, and Rad. Following that, a prediction model for the conversion of CU to CI was established using main queues. The backward stepwise selection method was employed using the likelihood ratio test and AIC as criteria for selecting the optimal predictive model [53]. Using the “rms” package in R, a predictive nomogram for prognosis combining the aforementioned 6 indicators was created.

To validate the optimal model externally, we performed the ROC curve analysis and calibration curve analysis using the dataset from the ADNI cohort. In addition, a Delong test was applied to assess the statistical significance of the difference in AUC values between the SILCODE cohort (training dataset) and the ADNI cohort (validation dataset).

### Clinical performance of the model

We compared the efficacy of various combinations of single predictors using the following approaches in order to validate the usefulness of the aforementioned model in clinical settings.

The predictive power of the indicators was assessed using AUC, and the implementation of decision curve analysis (DCA) allowed for the quantification of net benefits at various threshold probabilities. Both SILCODE and ADNI cohorts were included for external validation. Additionally, general linear model repeated correlation analysis, controlling for covariates (i.e., age, gender, and education), was conducted to make sure that the associations between blood markers, Rad, clinical information, and plasma biomarkers were not influenced by these covariates.

### Statistical analysis

The categorical variables (gender and APOEε4 carrier status) from demographic and neuropsychological data were summarized and displayed as percentages, and evaluated using the chi-square (*χ*^2^) test to determine group difference. Continuous variables, such as age and education level, calculated as means ± standard deviations, were compared using the independent 2-sample *t* test. The Kaplan–Meier method was used to build the survival curves to forecast the likelihood and timing of the conversion, and the log-rank test (survminer R package) was used for further comparison. Conducting mediation analysis (utilizing R; Lavaan package) was the final step to investigate the sequential relationships among the longitudinal changes of the Rad score, plasma Aβ42/Aβ40, p-tau181, NfL, and GFAP. The above statistical analyses were performed in R version 4.1.3 (http://www.r-project.org/), and the significance threshold was set at *P* < 0.05.

### Ethics approval, consent to participate, and consent for publication

This study was approved by the Medical Ethics Committee of Xuanwu Hospital, Capital Medical University, and was conducted in accordance with the Helsinki Declaration. All participants provided written informed consent and authorized the publication of their clinical details. SILCODE is listed on the ClinicalTrials.gov registry (SILCODE: NCT03370744). The authors take complete responsibility for the data, the analyses and interpretation, and the conduct of the research. They had unrestricted access to all of the data and possess the authority to publish any and all data separate and apart from any sponsor.

## Data Availability

The data used to support the findings of this study are available from the corresponding authors upon request.
