# Supplementary material for: Innovative Multivariable Model Combining MRI Radiomics and Plasma Indexes Predicts Alzheimer’s Disease Conversion: Evidence from a 2-Cohort Longitudinal Study
Source: Research (Wash D C). 2024 Apr 16;7:0354. doi: 10.34133/research.0354 (PMC11070845; doi:10.34133/research.0354)
Supplement: Supplementary 1 — Figs. S1 to S12 [file research.0354.f1.zip › supplementary material R2.docx]

**Innovative Multi-Variable Model Combining MRI Radiomics and Plasma Indexes Predicts Alzheimer's Disease Conversion: Evidence from a Two-Cohort Longitudinal Study**

**Supplementary material**

**Supplemental Methods**

MRI imaging processing

**Supplemental Figures and Tables**

Figures S1-16, Tables S1-17

**Supplemental References**

**Supplemental Methods**

**MRI radiomics processing**

**T1 image preprocessing：**

1. Converts dicom (Digital Imaging and Communications in Medicine) file to Nifi (Neuroimaging Informatics Technology Initiative) file with y_Call_Dcm2nii function in the DPABI V6.1 toolkit.

（2）Use SPM12 to segment T1 images into gray matter, white matter, cerebrospinal fluid tissue probability map, skull, and other tissues (corresponding to c1-c5)。

（3）Standardize the segmented cortical image (c1) space to MNI space, with a voxel size of 2mm×2mm×2mm。

（4）Use 8mm×8mm×8mm isotropic Gaussian smoothing kernel for smoothing。

**Computations of radiomics features：**

The radiomics features calculated here are based on the prior brain atlas AAL^[1]^, and the radiomics features of the 1-90 brain regions are extracted. Each brain region contains 43 features (in the order of 1-43 labels in the text), and each subject has 43 * 90=3870 features.

The data distribution in the table is as follows：

| ID | No.1 brain region | | | | No.2 brain region | | | | No.3 brain region | | | |
| --- | --- | --- | --- | --- | --- | --- | --- | --- | --- | --- | --- | --- |
| 1 | feature1 | feature2 | … | feature43 | feature1 | feature2 | … | feature43 | feature1 | feature2 | … | feature43 |
| 2 | feature1 | feature2 | … | feature43 | feature1 | feature2 | … | feature43 | feature1 | feature2 | … | feature43 |
| 3 | feature1 | feature2 | … | feature43 | feature1 | feature2 | … | feature43 | feature1 | feature2 | … | feature43 |

The tool kit used here is available at <https://github.com/mvallieres/radiomics/>。

**90 Brain regions are shown below**

| Index Number | Full name | abbreviation |
| --- | --- | --- |
| 1 | Precental gyrus | PreCG.L |
| 2 | Precental gyrus | PreCG.R |
| 3 | Superior frontal gyrus, dorsolateral | SFGdor.L |
| 4 | Superior frontal gyrus, dorsolateral | SFGdor.R |
| 5 | Superior frontal gyrus, orbital part | ORBsup.L |
| 6 | Superior frontal gyrus, orbital part | ORBsup.R |
| 7 | Middle frontal gyrus | MFG.L |
| 8 | Middle frontal gyrus | MFG.R |
| 9 | Middle frontal gyrus, orbital part | ORBmid.L |
| 10 | Middle frontal gyrus, orbital part | ORBmid.R |
| 11 | Inferior frontal gyrus, opercular part | IFGoperc.L |
| 12 | Inferior frontal gyrus, opercular part | IFGoperc.R |
| 13 | Inferior frontal gyrus, triangular part | IFGtriang.L |
| 14 | Inferior frontal gyrus, triangular part | IFGtriang.R |
| 15 | Inferior frontal gyrus, orbital part | ORBinf.L |
| 16 | Inferior frontal gyrus, orbital part | ORBinf.R |
| 17 | Rolandic operculum | ROL.L |
| 18 | Rolandic operculum | ROL.R |
| 19 | Supplementary motor area | SMA.L |
| 20 | Supplementary motor area | SMA.R |
| 21 | Olfactory cortex | OLF.L |
| 22 | Olfactory cortex | OLF.R |
| 23 | Superior frontal gyrus, medial | SFGmed.L |
| 24 | Superior frontal gyrus, medial | SFGmed.R |
| 25 | Superior frontal gyrus, medial orbital | ORBsupmed.L |
| 26 | Superior frontal gyrus, medial orbital | ORBsupmed.R |
| 27 | Gyrus rectus | REC.L |
| 28 | Gyrus rectus | REC.R |
| 29 | Insula | INS.L |
| 30 | Insula | INS.R |
| 31 | Anterior cingulate and paracingulate gyri | ACG.L |
| 32 | Anterior cingulate and paracingulate gyri | ACG.R |
| 33 | Median cingulate and paracingulate gyri | DCG.L |
| 34 | Median cingulate and paracingulate gyri | DCG.R |
| 35 | Posterior cingulate gyrus | PCG.L |
| 36 | Posterior cingulate gyrus | PCG.R |
| 37 | Hippocampus | HIP.L |
| 38 | Hippocampus | HIP.R |
| 39 | Parahippocampal gyrus | PHG.L |
| 40 | Parahippocampal gyrus | PHG.R |
| 41 | Amygdala | AMYG.L |
| 42 | Amygdala | AMYG.R |
| 43 | Calcarine fissure and surrounding cortex | CAL.L |
| 44 | Calcarine fissure and surrounding cortex | CAL.R |
| 45 | Cuneus | CUN.L |
| 46 | Cuneus | CUN.R |
| 47 | Lingual gyrus | LING.L |
| 48 | Lingual gyrus | LING.R |
| 49 | Superior occipital gyrus | SOG.L |
| 50 | Superior occipital gyrus | SOG.R |
| 51 | Middle occipital gyrus | MOG.L |
| 52 | Middle occipital gyrus | MOG.R |
| 53 | Inferior occipital gyrus | IOG.L |
| 54 | Inferior occipital gyrus | IOG.R |
| 55 | Fusiform gyrus | FFG.L |
| 56 | Fusiform gyrus | FFG.R |
| 57 | Postcentral gyrus | PoCG.L |
| 58 | Postcentral gyrus | PoCG.R |
| 59 | Superior parietal gyrus | SPG.L |
| 60 | Superior parietal gyrus | SPG.R |
| 61 | Inferior parietal, but supramarginal and angular gyri | IPL.L |
| 62 | Inferior parietal, but supramarginal and angular gyri | IPL.R |
| 63 | Supramarginal gyrus | SMG.L |
| 64 | Supramarginal gyrus | SMG.R |
| 65 | Angular gyrus | ANG.L |
| 66 | Angular gyrus | ANG.R |
| 67 | Precuneus | PCUN.L |
| 68 | Precuneus | PCUN.R |
| 69 | Paracentral lobule | PCL.L |
| 70 | Paracentral lobule | PCL.R |
| 71 | Caudate nucleus | CAU.L |
| 72 | Caudate nucleus | CAU.R |
| 73 | Lenticular nucleus, putamen | PUT.L |
| 74 | Lenticular nucleus, putamen | PUT.R |
| 75 | Lenticular nucleus, pallidum | PAL.L |
| 76 | Lenticular nucleus, pallidum | PAL.R |
| 77 | Thalamus | THA.L |
| 78 | Thalamus | THA.R |
| 79 | Heschl gyrus | HES.L |
| 80 | Heschl gyrus | HES.R |
| 81 | Superior temporal gyrus | STG.L |
| 82 | Superior temporal gyrus | STG.R |
| 83 | Temporal pole: superior temporal gyrus | TPOsup.L |
| 84 | Temporal pole: superior temporal gyrus | TPOsup.R |
| 85 | Middle temporal gyrus | MTG.L |
| 86 | Middle temporal gyrus | MTG.R |
| 87 | Temporal pole: middle temporal gyrus | TPOmid.L |
| 88 | Temporal pole: middle temporal gyrus | TPOmid.R |
| 89 | Inferior temporal gyrus | ITG.L |
| 90 | Inferior temporal gyrus | ITG.R |

The following is the meaning and calculation method of each feature：

**Features based on intensity histogram (first-order gray feature)**

The image gray histogram quantifies the global heterogeneity of the image region by measuring the occurrence frequency of each gray level without considering the correlation between pixels. Therefore, the features based on the gray histogram cannot reflect the spatial statistical relationship between image pixels.

The features based on the gray histogram can quantitatively describe the heterogeneity of image regions. Assuming that it represents the number of different gray levels, p(i) represents the number of pixels in the normalized gray histogram (the number of pixels in each gray level divided by the total number of pixels in the ROI). Features based on intensity histogram include:

**1.**$\mathbf{Variance}$**：**Describe the change of intensity near the mean value, thus indicating the level of heterogeneity in the region.

|  | $Variance=\sigma^{2}=\sum_{i=1}^{N_{g}} (i-\mu)^{2}p(i)$ | (2-4) |
| --- | --- | --- |

**2.**$\boldsymbol{Skewness}$**：**Measure the asymmetry of the histogram. If the histogram is symmetrical about the mean value, the feature is zero, and if it is skewed above or below the mean value, the feature is positive or negative.

|  | $Skewness=\sigma^{-3}\sum_{i=1}^{N_{g}} (i-\mu)^{3}p(i)$ | (2-5) |
| --- | --- | --- |

**3.**$\boldsymbol{Kurtosis}$**：**Flatness (positive) or peak (negative) of measured histogram compared with normal distribution.

|  | $Kurtosis =\sigma^{-4}\sum_{i=1}^{N_{g}} [(i-\mu)^{4}p(i)]-3$ | (2-6) |
| --- | --- | --- |

**Gray level co-occurrence matrix**

Gray level co-occurrence matrix (GLCM) is a second-order statistical texture analysis method first proposed by Haralick et al., which is used to describe local heterogeneity information ^[2-5]^. This method quantifies the relationship between gray levels in an image region by counting the number of pixels with the same gray level distribution at a predetermined distance (𝑑) and direction ($\theta$) interval. The value in the resulting matrix represents the total number of gray levels of the reference pixel with a specific gray level in the image and the adjacent pixel (located at a distance of 𝑑 from the reference pixel and in the direction of$\theta$). Therefore, the matrix size of GLCM represents the number of different gray levels. Figure 1 shows a simple example of calculating GLCM.


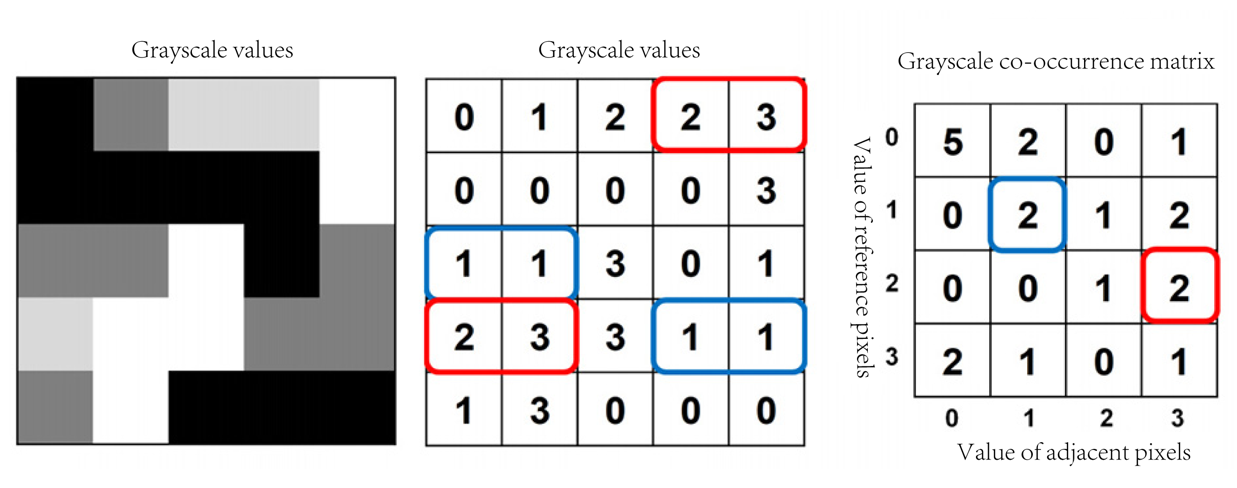


Figure 1 Use an example image (5*5 pixels) to calculate GLCM. The sample image contains 4 gray levels. In the horizontal direction ($\theta$=$0^{\circ}$) and a pixel spacing (𝑑 = 1), calculate GLCM. The blue and red values in GLCM represent the marked pixel pairs with the same gray level distribution (for example, take the pixel with gray level 2 as the reference pixel, and the pixel with the distance between the reference pixel and the reference pixel is 1, the direction is and the gray level is 3 as the adjacent pixel, such pixel pairs have 2 pairs in total, so the red value in GLCM is 2; and so on)

As mentioned earlier, GLCM depends on distance and direction, and is sensitive to the selection of distance and direction. For example, different texture values will be obtained when rotating an image. To solve this problem, we can calculate GLCM in four directions (horizontal, vertical, 45 °, 135 °) in two-dimensional space (13 directions in three-dimensional space), and then average or sum these matrices to obtain a single comprehensive matrix with rotation invariance. You can also calculate the GLCM in each spacing and direction, extract the corresponding GLCM features, and then calculate the average or sum.

Quantitative features can be extracted from GLCM by mathematical methods to quantify the image area’s uniformity (smoothness) or heterogeneity (roughness). $p(i,j)$represents the total logarithm of the reference pixel with the gray level and the adjacent pixel with the gray level in the image area in the normalized GLCM. $p_{x}\left( i \right)=\sum_{i=1}^{N_{\mathrm{rows}}} p\left( i,j \right)$and $p_{y}(j)=\sum_{i=1}^{N_{column}} p(i,j)$ represent the edge probability obtained by summing the row or column of the $i$ and $j$ entries in the normalized GLCM. $\mu_{x},\mu_{y},\sigma_{x},\sigma_{y}$ represents the mean and standard deviation of $p_{x}$ and $p_{y}$. $p_{x+y}(k)$ and $p_{x-y}(k)$ represent the probability of diagonal and cross diagonal, respectively representing the distribution of the gray sum ($k=i+j$) and gray difference ($k=|i-j|$). Based on the above definition, the following characteristics can be calculated from GLCM:

**4.** $\boldsymbol{Energy}$：Also known as second-order moment or uniformity, it is used to measure the local uniformity (or order) of the image. The higher value is related to the uniform region, indicating that the intensity in the region is very similar.

|  | $Energy =\sum_{i=1}^{N_{g}} \sum_{j=1}^{N_{g}} [p(i,j)]^{2}$ | (2-9) |
| --- | --- | --- |

1. $\mathbf{Contrast}$**：**Describe the local intensity changes between different structures in the image area. Higher values are associated with higher heterogeneity.

|  | $Contrast = \sum_{i=1}^{N_{g}} \sum_{j=1}^{N_{g}} (i-j)^{2}p(i,j)$ | (2-10) |
| --- | --- | --- |

**6.**$\boldsymbol{Entr}\boldsymbol{opy}$**：**Indicates the degree of chaos or disorder in the image (i.e. randomness). Higher values mean random distribution, i.e. heterogeneous regions.

|  | $Entropy =-\sum_{i=1}^{N_{g}} \sum_{j=1}^{N_{g}} p(i,j)log(p(i,j))$ | (2-15) |
| --- | --- | --- |

**7.**$\boldsymbol{Ho}\boldsymbol{mogeneity}$**：**Also known as the inverse moment, it describes the local homogeneity of the image. Higher values relate to smooth areas (most of which have the same gray level).

|  | $Homogeneity =\sum_{i=1}^{N_{g}} \sum_{j=1}^{N_{g}} \frac{p(i,j)}{1+(i-j)^{2}}$ | (2-12) |
| --- | --- | --- |

**8.**$\boldsymbol{Cor}\boldsymbol{relation}$**：**The gray linear correlation between the measured intensities represents how the reference pixels (or voxels) are related to their nearby parts. A higher value indicates that the correlation between pixels (or voxels) is high, that is, the homogeneity is strong.

|  | $Correlation=\sum_{i=1}^{N_{g}} \sum_{j=1}^{N_{g}} \frac{ijp(i,j)-\mu_{x}\mu_{y}}{\sigma_{x}\sigma_{y}}$ | (2-11) |
| --- | --- | --- |

**9.**$\boldsymbol{Sum}\boldsymbol{Average}$**：**Estimate the overall brightness of the image, and calculate the average value of all gray combinations.

|  | $Sum Average =\sum_{i=1}^{N_{g}} \sum_{j=1}^{N_{g}} [ip(i,j)+jp(i,j)]$ | (2-14) |
| --- | --- | --- |

**10.**$\boldsymbol{Varia}\boldsymbol{nce}$**：**Also known as the sum of squares, it is used to measure the dispersion of the gray distribution near the mean value, that is, the randomness of the pixel (or voxel) distribution in the image. Higher values refer to heterogeneous regions (gray value distribution is discrete).

|  | $Variance =\sum_{i=1}^{N_{g}} \sum_{j=1}^{N_{g}} (1-\mu)^{2}p(i,j)$ | (2-13) |
| --- | --- | --- |

**11.**$\boldsymbol{Dissi}\boldsymbol{milarity}$**：**Also known as difference averaging, it is used to measure the difference level between pixel (or voxel) pairs in an image.

|  | $Dissimilarity =\sum_{i=1}^{N_{g}} \sum_{j=1}^{N_{g}} \vert i-j\vert p(i,j)$ | (2-17) |
| --- | --- | --- |

**12.**$\boldsymbol{Au}\boldsymbol{tocorrelation}$**：**Measure the correlation between pixel (or voxel) pairs.

|  | $Autocorrelation =\sum_{i=1}^{N_{g}} \sum_{j=1}^{N_{g}} ijp(i,j)$ | (2-16) |
| --- | --- | --- |

**Gray run matrix**

Gray-level run-length matrix (GLRLM) is a high-order texture analysis method based on the statistical matrix, which is used to describe regional heterogeneity information^[6-9]^. GLRLM is constructed by detecting and counting the runs of different gray levels (sequences of consecutive pixels with the same gray level) and their lengths in the image. Each row of GLRLM represents a gray level, and each column represents a specific length. Therefore, each matrix element represents the number of runs of a specific gray level and length in the image. GLRLM is sensitive to direction. In order to achieve rotation invariance, the method of re-averaging GLRLM in all directions is usually adopted, as previously described for GLCM. Generally, the features extracted from GLRLM can be used to define fine texture (mainly short run) or rough texture (mainly long run). $N_{g}$ represents the number of different gray levels, and $N_{r}$ represents the run of the maximum distance. In the normalized GLRLM, the element $r(i,j)$ represents the number of runs with the gray level of $i$ and the run distance of $j$. Based on GLRLM, the following characteristics can be defined:

**13.Short Run Emphasis, SRE：**Emphasis on short-distance travel.

|  | $SRE=\sum_{i=1}^{N_{g}} \sum_{j=1}^{N_{r}} \frac{r(i,j)}{j^{2}}$ | (2-18) |
| --- | --- | --- |

**14.Long Run Emphasis(LRE）**：Emphasis on long-distance travel.

|  | $LRE=\sum_{i=1}^{N_{g}} \sum_{j=1}^{N_{r}} j^{2}r(i,j)$ | (2-19) |
| --- | --- | --- |

**15.Gray-level Non-uniformity, GLN**：Evaluate the run distribution with the gray level as the reference. The lower value is related to the approximate uniform distribution of the run relative to the gray level.

|  | $GLN=\sum_{i=1}^{N_{g}} {(\sum_{j=1}^{N_{r}} r\left( i,j \right))}^{2}$ | (2-20) |
| --- | --- | --- |

**16.Run-Length Non-uniformity, RLN**：The lower value is related to the approximate uniform distribution of run relative to run length.

|  | $RLN=\sum_{j=1}^{N_{r}} {(\sum_{i=1}^{N_{g}} r\left( i,j \right))}^{2}$ | (2-21) |
| --- | --- | --- |

**17.Run Percentage, RP：**Evaluate the ratio between the actual number of trips and the maximum number of possible trips. The lower values are related to areas with strong linearity or high uniformity.

|  | $RP=\frac{\sum_{i=1}^{N_{g}} \sum_{j=1}^{N_{r}} r(i,j)}{\sum_{i=1}^{N_{g}} j\sum_{j=1}^{N_{r}} r(i,j)}$ | (2-22) |
| --- | --- | --- |

**18.Low Gray-level Run Emphasis, LGRE：**Similar to SRE, but the emphasis is on low-gray runs rather than short runs.

|  | $LGRE=\sum_{i=1}^{N_{g}} \sum_{j=1}^{N_{r}} \frac{r(i,j)}{i^{2}}$ | (2-23) |
| --- | --- | --- |

**19.High Gray-level Run Emphasis, HGRE：**Similar to LRE, but the emphasis is on the high-gray range rather than the long-distance range.

|  | $HGRE=\sum_{i=1}^{N_{g}} \sum_{j=1}^{N_{r}} i^{2}r(i,j)$ | (2-24) |
| --- | --- | --- |

**20.Short Run Low Gray-level Emphasis, SRLGE**：Highlight the middle run in the upper left quadrant of GLRLM, where the short run length and low gray level are located in this quadrant.

|  | $SRLGE=\sum_{i=1}^{N_{g}} \sum_{j=1}^{N_{r}} \frac{r(i,j)}{i^{2}j^{2}}$ | (2-25) |
| --- | --- | --- |

**21.Short Run High Gray-level Emphasis, SRHGE：**Highlight the middle run in the lower left quadrant of GLRLM, where the short run length and high gray level are located in this quadrant.

|  | $SRHGE=\sum_{i=1}^{N_{g}} \sum_{j=1}^{N_{r}} \frac{i^{2}r(i,j)}{j^{2}}$ | (2-26) |
| --- | --- | --- |

**22.Long Run Low Gray-level Emphasis, LRLGE：**Highlight the middle run in the upper right quadrant of GLRLM, where the long run length and low gray level are located in this quadrant.

|  | $LRLGE=\sum_{i=1}^{N_{g}} \sum_{j=1}^{N_{r}} \frac{j^{2}r(i,j)}{i^{2}}$ | (2-27) |
| --- | --- | --- |

**23.Long Run High Gray-level Emphasis, LRHGE：**Highlight the middle run in the lower right quadrant of GLRLM, where the long run length and high gray level are located in this quadrant.

|  | $LRHGE=\sum_{i=1}^{N_{g}} \sum_{j=1}^{N_{r}} {i^{2}j}^{2}r(i,j)$ | (2-28) |
| --- | --- | --- |

**24.Gray-level Variance, GLV：**Estimate the variance of the run distribution based on the gray level.

|  | $GLV=\frac{1}{N_{g}\times N_{r}}\sum_{i=1}^{N_{g}} \sum_{j=1}^{N_{r}} (ir(i,j){-\sum_{i=1}^{N_{g}} i\sum_{j=1}^{N_{r}} r(i,j))}^{2}$ | (2-29) |
| --- | --- | --- |

**25.Run-Length Variance, RLV：**Estimate the variance of the run distribution based on the run length.

|  | $RLV=\frac{1}{N_{g}\times N_{r}}\sum_{i=1}^{N_{g}} \sum_{j=1}^{N_{r}} (jr(i,j){-\sum_{j=1}^{N_{r}} j\sum_{i=1}^{N_{g}} r(i,j))}^{2}$ | (2-30) |
| --- | --- | --- |

**Grayscale area size matrix**

Grayscale region size matrix (GLSZM) is a texture analysis method based on the higher-order statistical matrix proposed by Thibault et al. in 2009 ^[10-13]^. It describes the heterogeneity information in the image region similarly to GLRLM. The concept of GLSZM is to extend the run to an area or volume based on GLRLM. In this way, GLSZM calculates the number of regions with a specific size and the same gray level (that is, connected regions with the same gray level) in the entire image. The calculation example of this matrix is shown in Figure 2.
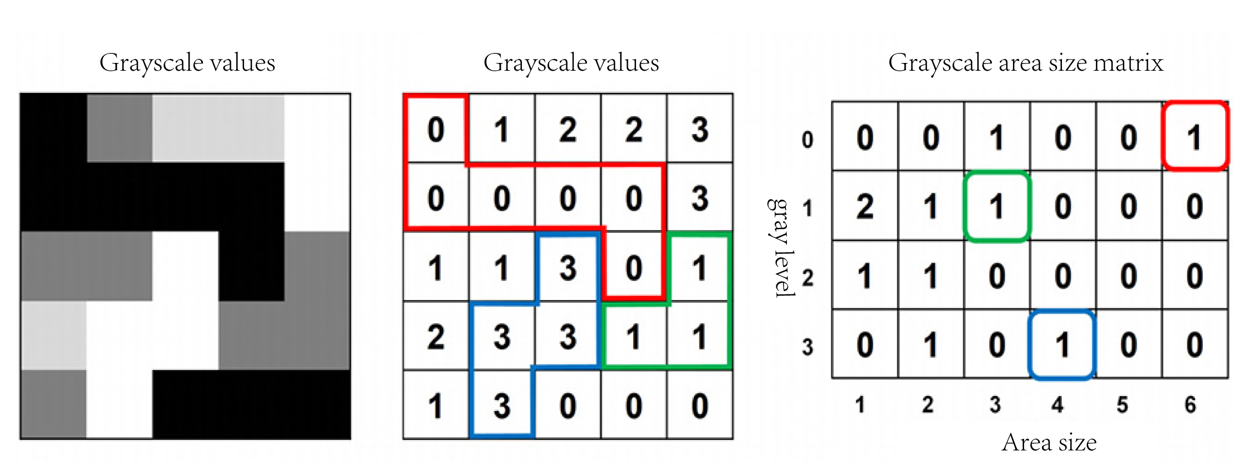


Figure 2 Use an example image (5 × 5 pixels) to calculate GLSZM, which contains 4 gray levels. The calculation of GLSZM does not depend on distance or direction and has rotation invariance. Only one GLSZM can be calculated for each image. The blue, red, and green values represent the number of areas (connected areas with the same gray level) with a specific size and gray level found in the image.

The advantage of GLSZM is that its calculation is independent of direction and distance and has rotation invariance. Only one GLSZM can be calculated for each image region. The features extracted from GLSZM are similar to those defined for GLRLM (Formula 2-18 to Formula 2-30), but the number of areas ($N_{z}$) is used instead of the number of trips ($N_{r}$). Therefore, the features extracted from GLSZM include:

**26.Small Zone Emphasis(SZE)，**

**27.Large Zone Emphasis(LZE)，**

**28.Gray-level Non-uniformity(GLN)，**

**29.Zone-Size Non-uniformity(ZSN)，**

**30.Zone Percentage(ZP)，**

**31.Low Gray-level Zone Emphasis(LGZE)，**

**32.High Gray-level Zone Emphasis(HGZE)，**

**33.Small Zone Low Gray-level Emphasis(SZLGE)，**

**34.Small Zone High Gray-level Emphasis(SZHGE)，**

**35.Large Zone Low Gray-level Emphasis(LZLGE)，**

**36.Large Zone High Gray-level Emphasis(LZHGE)，**

**37.Gray-level Variance(GLV)，**

**38.Zone-Size Variance(ZSV)。**

**Gray neighborhood difference matrix**

The gray neighborhood difference matrix (NGTDM) is a high-order statistical texture analysis method used to describe the local heterogeneity of the analyzed image and was proposed by Amadasun and King in 1989 ^[14]^. It is an alternative method of GLCM. The purpose is to quantify the local attributes of the image by calculating the sum of the gray level difference between the pixel (or voxel) with gray level 𝑖 and the average value of the gray level of the adjacent pixel (or voxel) within the distance 𝑑 [70]. Therefore, entry 𝑖 of the NGTDM represents the sum of the differences between the average gray values of all voxels with gray level 𝑖 and their surrounding neighborhood (the pixel within the distance 𝑑 from the pixel with gray level 𝑖 is its neighborhood). As shown in Figure 3.


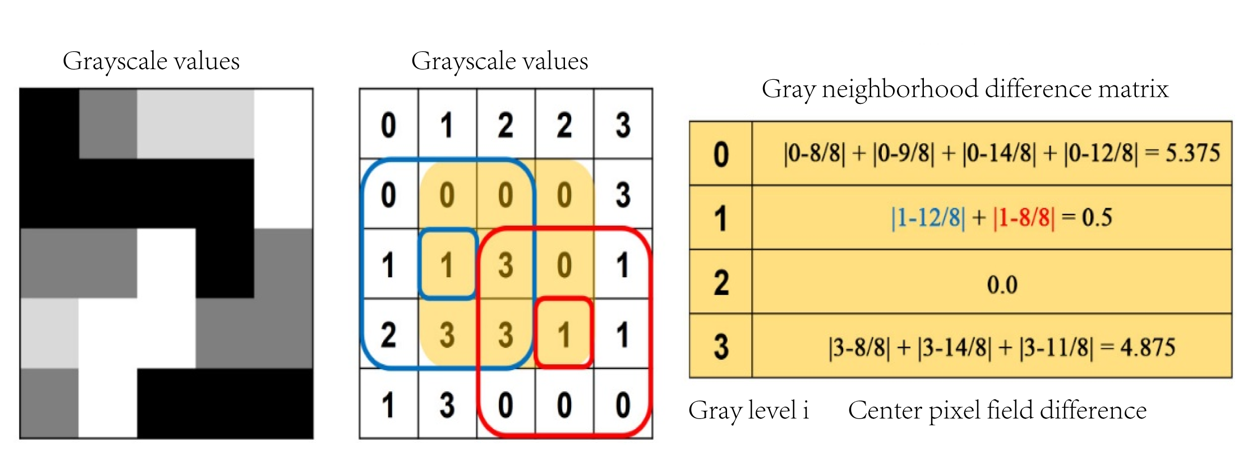


Figure 3 Select distance 𝑑=1, use example image (5×5 pixels). The sample image contains 4 gray levels. The orange square represents the neighborhood (distance 𝑑=1) of the central pixel (gray value is 3) of the image, and the non-orange area represents the peripheral area; The blue and red boxes represent the neighborhood of gray level 𝑖=1 pixel in the orange square. The gray level contained in the neighborhood of the central pixel (gray level value is 3) is 0,1,3; It does not contain 2 $P\left( i=2 \right)=0$; Take calculation $P\left( i=1 \right)$ as an example, there are two pixels with gray level 2 in the neighborhood of the central pixel (gray level value is 3), and their neighborhood corresponds to the blue box and the red box respectively.The gray level of the blue box is 1 pixel, and there are 8 neighboring pixels in total. The average gray level is (0+0+0+3+3+2+1)/8=8/8, so the difference between the gray level of the blue box is 1 pixel and the average gray level of their surrounding neighborhood is | 1-8/8 |, which corresponds to the blue formula in the right figure. The calculation of the red box is similar. The calculation of other gray levels in the neighborhood of the central pixel (gray level value is 3) and so on.

The NGTDM of a specific pixel is not actually a matrix, but an array, because its size is $N_{g}$×1, $N_{g}$ represents the highest gray level in the image. In addition, the calculation of NGTDM depends on distance but not on direction, so the default case (distance 𝑑=1) has rotation invariance. If 𝑠(𝑖) represents the 𝑖 the entry in NGTDM, it is defined as:

|  | $s(i)=\left\{ \begin{aligned} \begin{matrix} \sum_{0} \vert i-\overline{A}_{l}\vert& for i\in N_{i} if N_{i}\neq0, \\ & \end{matrix} \\ 0 otherwise. \end{aligned} \right.$ | (2-31) |
| --- | --- | --- |

Where, $N_{i}$ represents all sets in the image with a gray level of $i$ pixels (excluding peripheral areas when width is 𝑑), $\overline{A}_{l}$ represents the gray level average of neighborhood pixels located in a location $(k,l)$ with gray level of $i$ pixels, and $\overline{A}_{l}$ is represented as follows:

|  | $\overline{A_{i}}=\overline{A}(k,l)=\frac{1}{W-1}[\sum_{m=-d}^{d} \sum_{n=-d}^{d} f(k+m,l+n)],(m,n)\neq(0,0)$ | (2-32) |
| --- | --- | --- |

Where, 𝑑 represents the agreement with the selected distance in determining the neighborhood size, ${W=(2d+1)}^{2}$. Figure 3.9 also shows an example of how this matrix is calculated. Figure 5 shows an example of how this matrix is calculated.

In general, 5 features can be extracted from ngtdm. Assuming an image size of $N\times N$, the occurrence probability of gray value 𝑖 was defined as $n_{i}=\frac{N_{i}}{n^{2}}$, in which 5 extracted features in $n^{2}=N-2d$, NGTDM were defined as follows:

**39.**$\boldsymbol{Coarseness}$**：**The level of spatial rate of change in intensity is indicated, with higher values indicating a coarse texture with little difference in gray levels.

|  | $Coarseness ={[\epsilon+\sum_{i=1}^{N_{g}} n_{i}s(i)]}^{-1}$ | (2-34) |
| --- | --- | --- |

Where, 𝜖 represents a decimal constant to prevent this parameter from becoming infinitesimal.

**40.**$\boldsymbol{Contrast}$**：**Represents the contrast of the image, and higher values indicate a large difference in intensity between adjacent regions.

|  | $\text{ }Contrast =[\frac{1}{N_{g}^{eff}[N_{g}^{eff}-1]}\sum_{i=1}^{N_{g}} \sum_{j=1}^{N_{g}} n_{i}n_{j}(i-j)^{2}][\frac{1}{n^{2}}\sum_{i=1}^{N_{g}} s(i)]$ | (2-33) |
| --- | --- | --- |

Where $N_{g}^{eff}$ represents the effective number of different gray levels in the image (some gray levels may not appear in the image area due to the quantification process).

**41.**$\boldsymbol{Busyness}$**：**Representing the spatial frequency level of intensity change, higher values represent frequently changing textures (i.e., fast changes in intensity from one pixel to its neighboring pixels).

|  | $Busyness=\frac{\sum_{i=1}^{N_{g}} n_{i}s(i)}{\sum_{i=1}^{N_{g}} \sum_{j=1}^{N_{g}} (in_{i}-jn_{j})},n_{i}\neq0,n_{j}\neq0$ | (2-35) |
| --- | --- | --- |

**42.**$\boldsymbol{Complexity}$：The content of visual information is included in fingerprinting, and higher values indicate a texture complex, i.e., a non-uniform texture with a high information content, e.g., when there are many elementary units or patches.

|  | $Complexity =\sum_{i=1}^{N_{g}} \sum_{j=1}^{N_{g}} \frac{\vert i-j\vert[n_{i}s(i)+n_{j}s(j)]}{n^{2}(n_{i}+n_{j})},n_{i}\neq0,n_{j}\neq0$ | (2-36) |
| --- | --- | --- |

**43.**$\boldsymbol{Strength}$**：**Giving the concept of the degree of detail of the elementary units in the diagram, higher values are related to a strong texture in which the elementary units are easily defined and clearly visible.

|  | $Strength=\frac{\sum_{i=1}^{N_{g}} \sum_{j=1}^{N_{g}} (n_{i}+n_{j}){(i-j)}^{2}}{[\epsilon+\sum_{i=1}^{N_{g}} s(i)]},n_{i}\neq0,n_{j}\neq0$ | (2-37) |
| --- | --- | --- |

**Supplemental Figures and Tables**

**
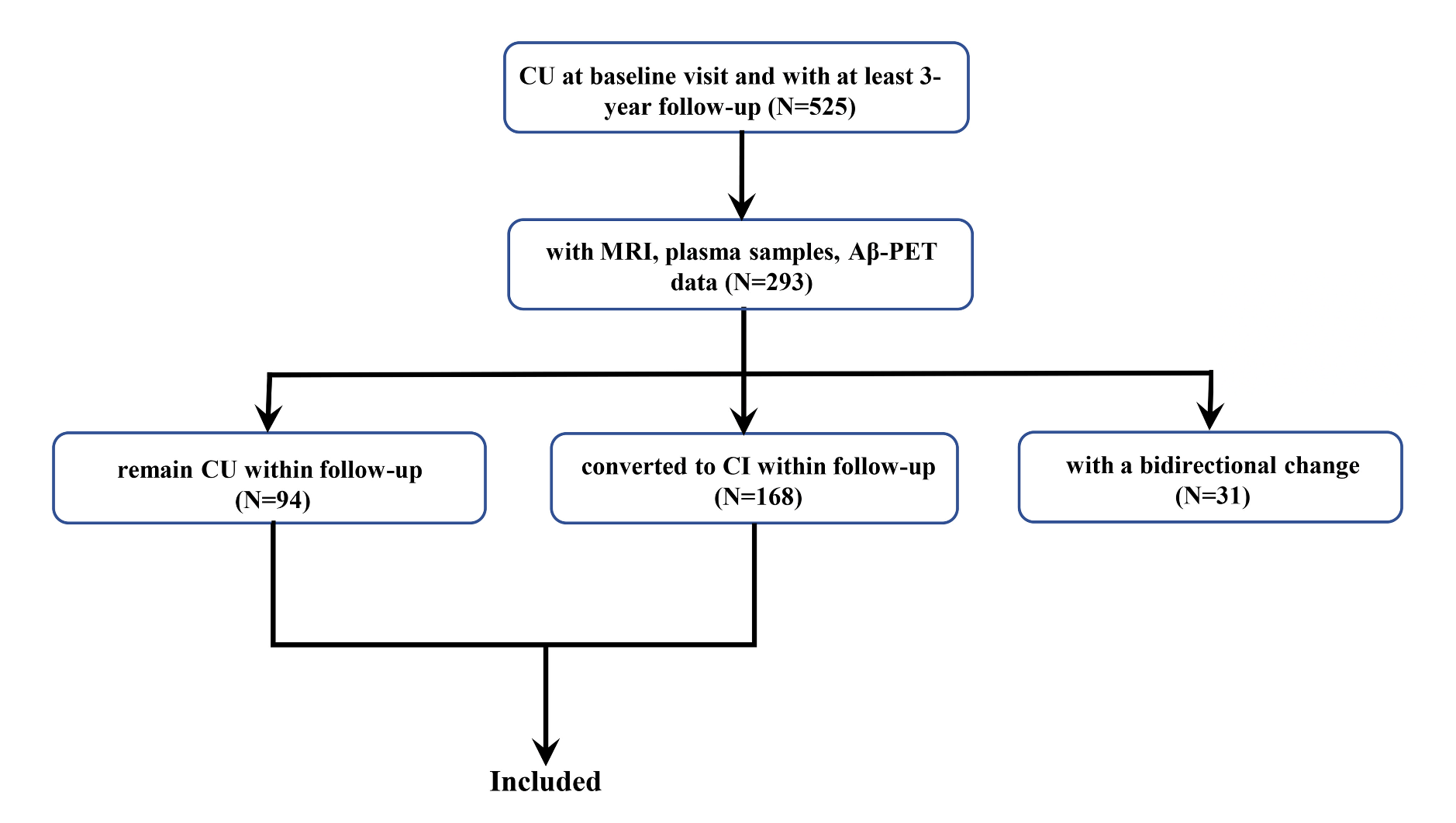
**

**Fig S1** Flow chart of enrollment of ADNI data. CU, cognitively unimpaired; CI, cognitive impairment.


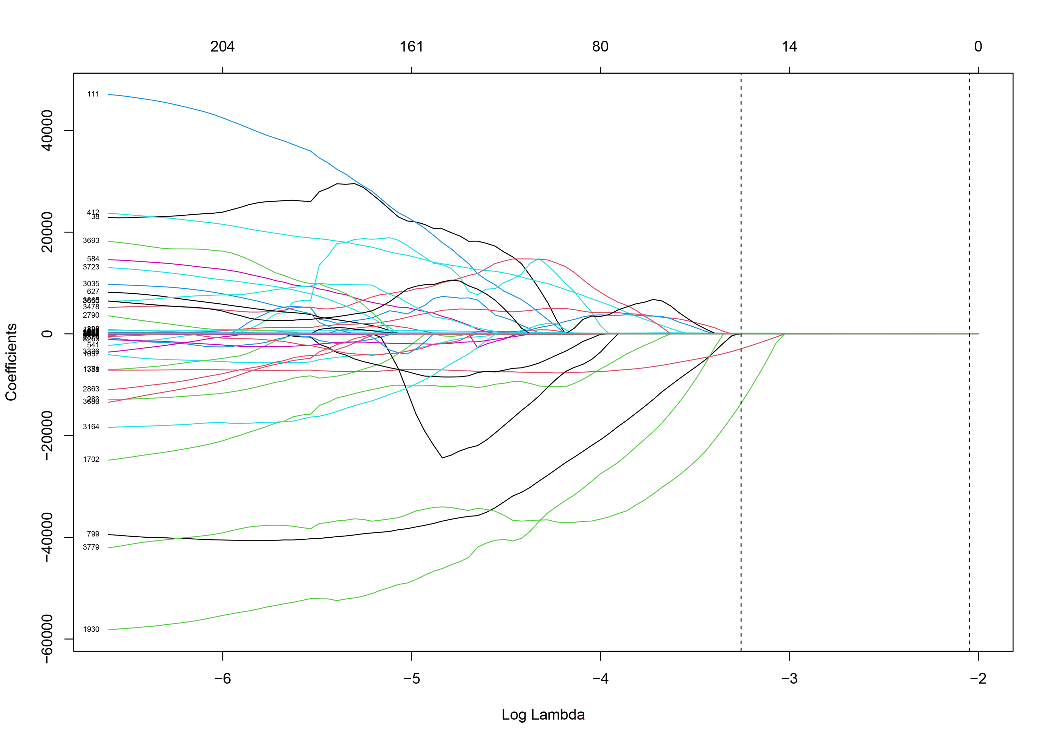
**
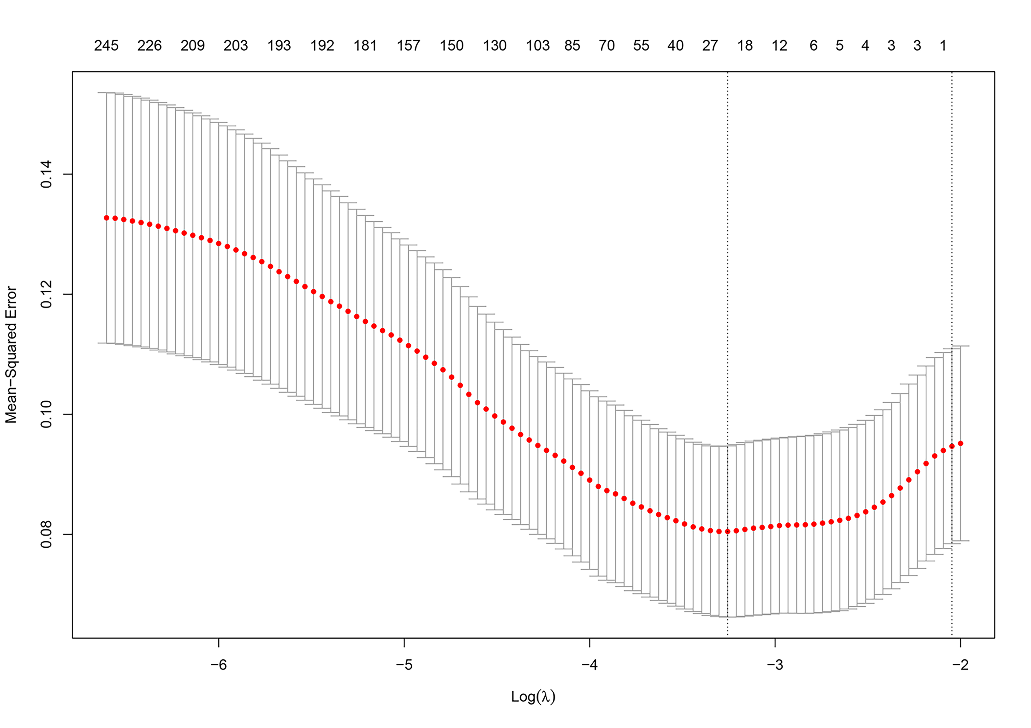
A B**

**Figure S2** Feature selection using the least absolute shrinkage and selection operator (LASSO) binary logistic regression model. (A) The tuning parameter (λ) in the LASSO model is chosen by the minimum criterion using 10-fold cross-validation. The graph shows the relationship between the area under the receiver operating characteristic (ROC) curve and log(λ). The vertical dotted lines are drawn at the optimal value based on the minimum criterion and the minimum standard error (1-SE criterion). (B) The LASSO coefficient profiles of 3870 texture features are plotted as a function of the logarithm of λ. The vertical line is drawn at the selected value based on 10-fold cross-validation, where the optimal λ produces 19 nonzero coefficients. Thirteen variables with P<0.05 are selected, and variables 37.13 and 37.16 did not pass the multicollinearity test. After analysis, we removed 37.13, and finally included 12 radiomics features.

**Figure S3** Pre-scores of the Rad for each patient.


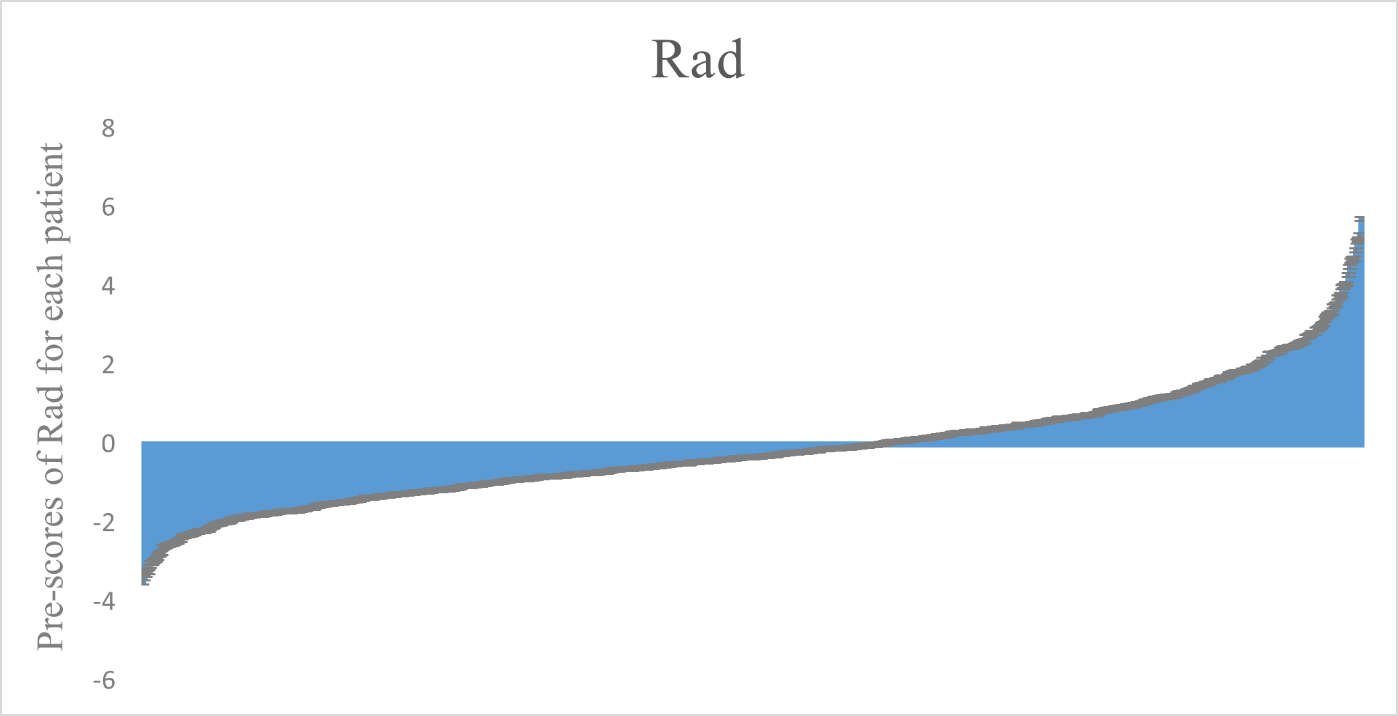


The following is the calculation formula for Rad-score.

Rad-score=201.2585+

(-0.00031* PreCG.L-𝐕𝐚𝐫𝐢𝐚𝐧𝐜𝐞) +

(1.68E-05* IFGoperc.R-SZHGE) +

(0.016842* INS.R-𝑺𝒕𝒓𝒆𝒏𝒈𝒕𝒉) +

(-2.7E-05*ACG.L-LZHGE) +

(19.88992*DCG.L-GLV) +

(-0.00077*PCG.R-𝐕𝐚𝐫𝐢𝐚𝐧𝐜𝐞) +

(-17.4704*PCG.R-SZE) +

(-178.506*HIP.L-RLN) +

(-3.4E-05*HIP.L-𝑪𝒐𝒎𝒑𝒍𝒆𝒙𝒊𝒕𝒚) +

(-0.45979*AMYG.R-SZE) +

(-0.00015*THA.L-LZHGE) +

(5.47E-05*ITG.L-SZHGE）

**Figure S4** Correlational analysis among 12 Rad features and plasma biomarkers.


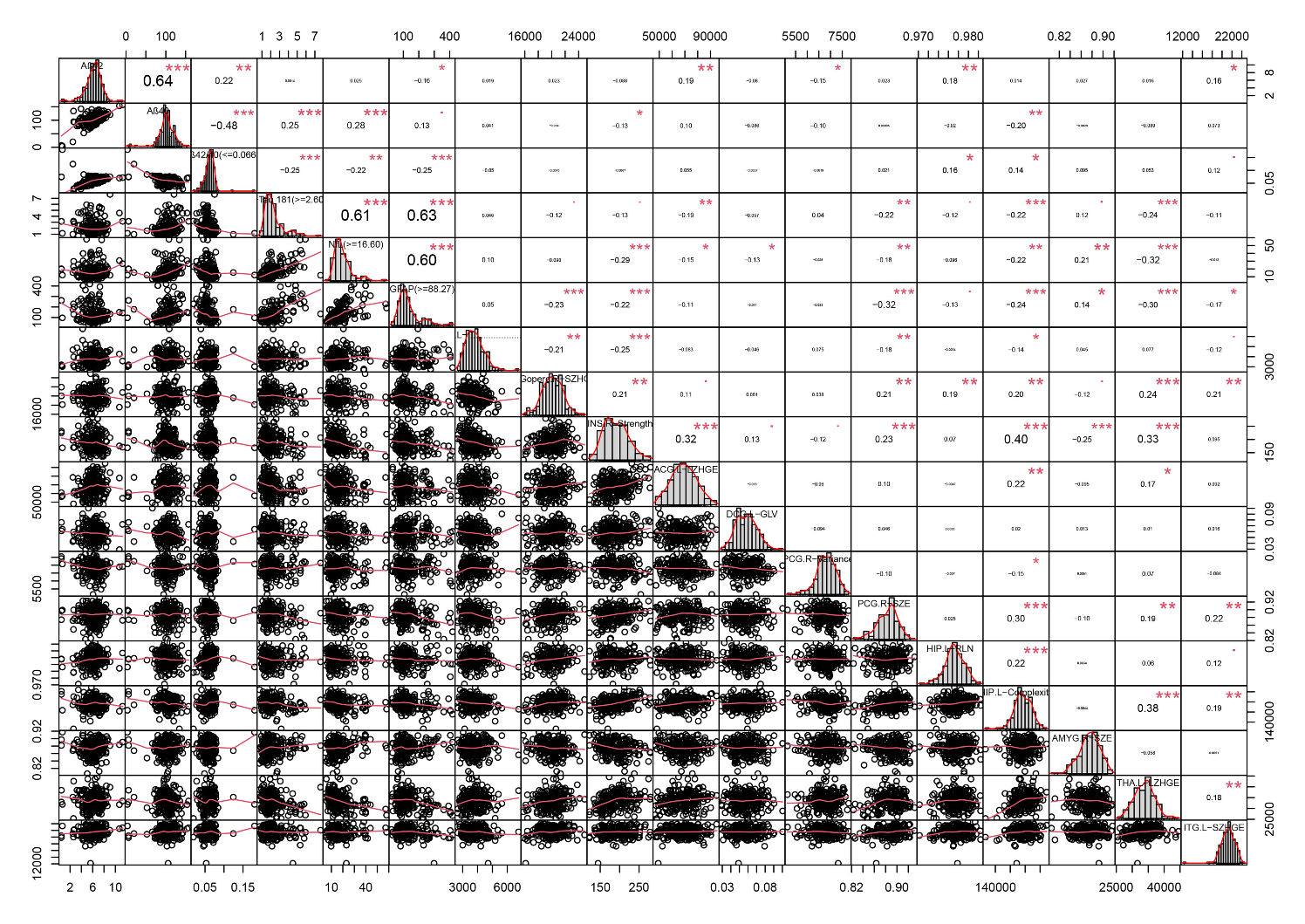


**Figure S5** The ROC curve and AUC of the Rad in the training and validation cohort. Abbreviations: AUC, area under the ROC curve; ROC, receiver operating characteristic.


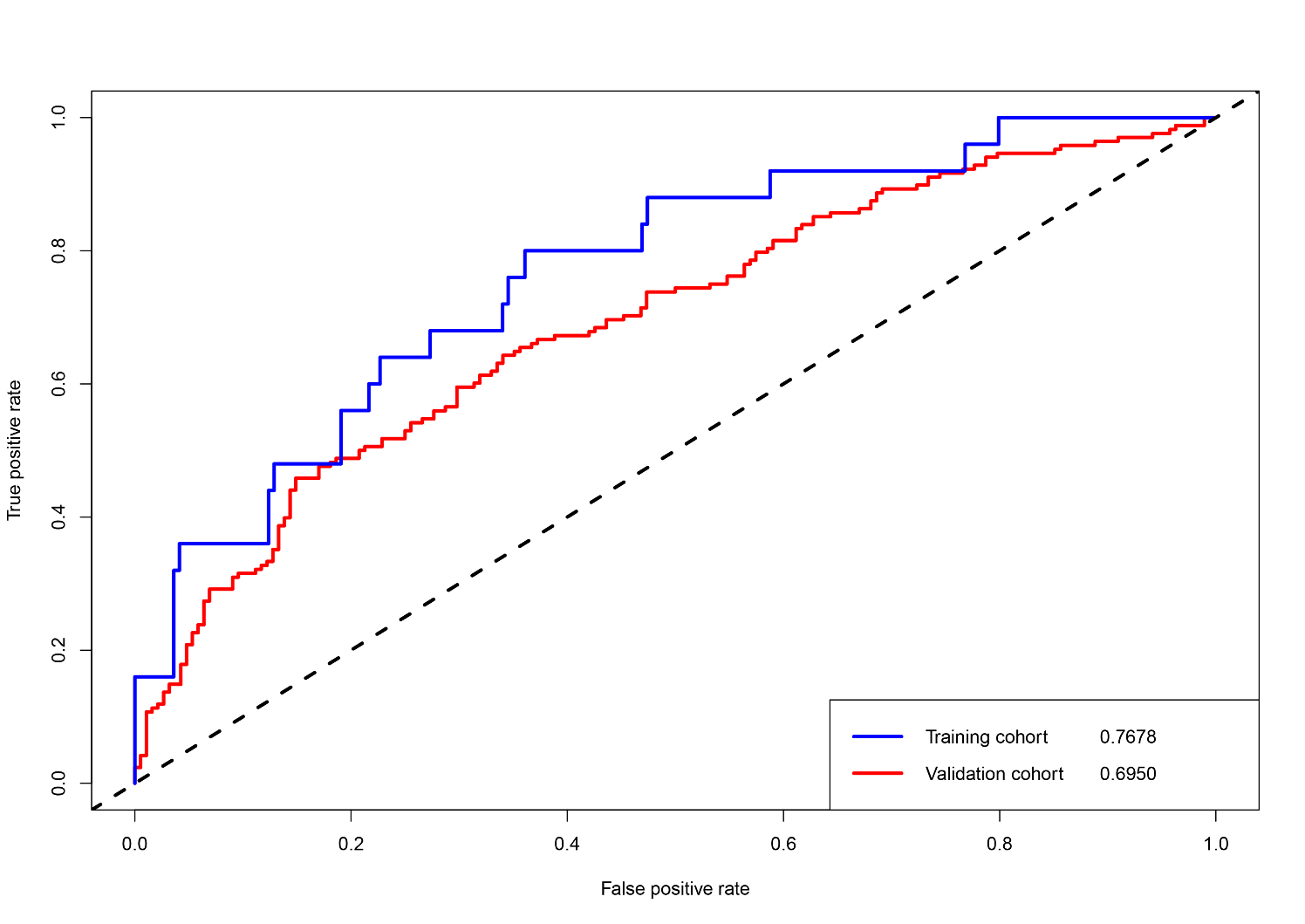


**Fig S6** Calibration plot of the Rad in the training and validation cohorts. The calibration plot for predicting prognosis conversion rate training cohort(A) and validation cohort(B). The actual rate of conversion is shown on the y-axis, and the Rad-predicted probability of conversion is shown on the x-axis.

**
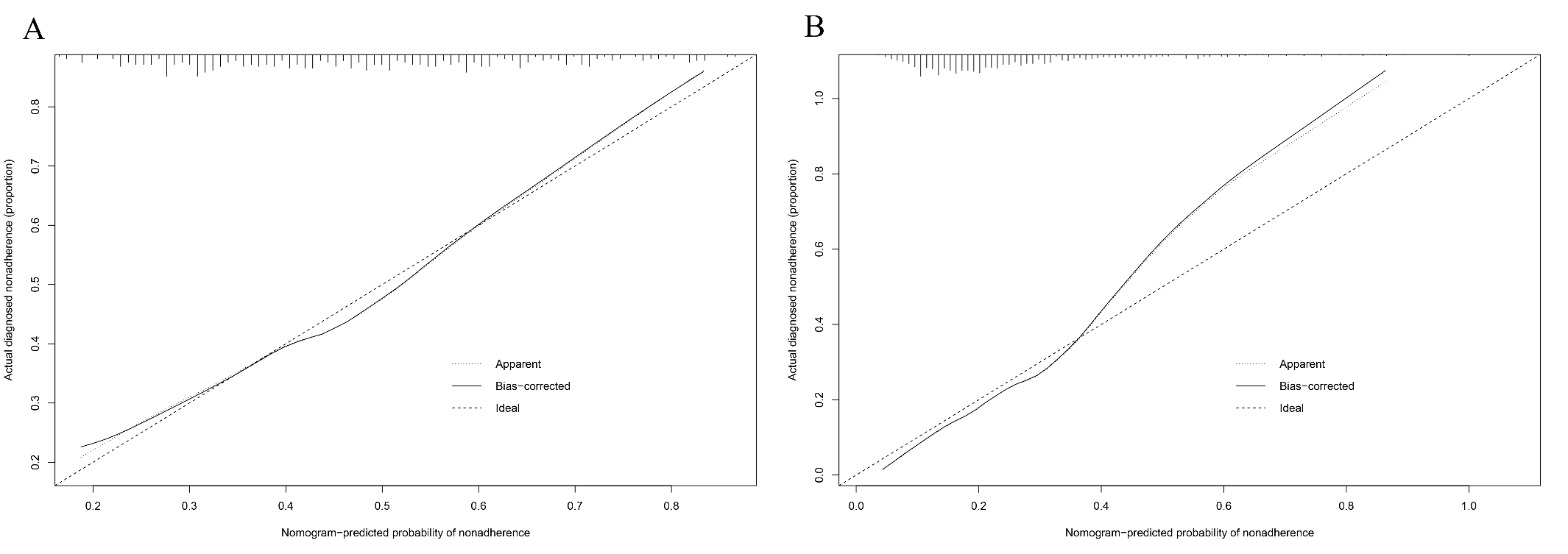
**

**Fig S7** The ROC curves of Aβ42/Aβ40, p-tau181, NfL (blue curve) and Aβ42/Aβ40, p-tau181, GFAP (red curve).


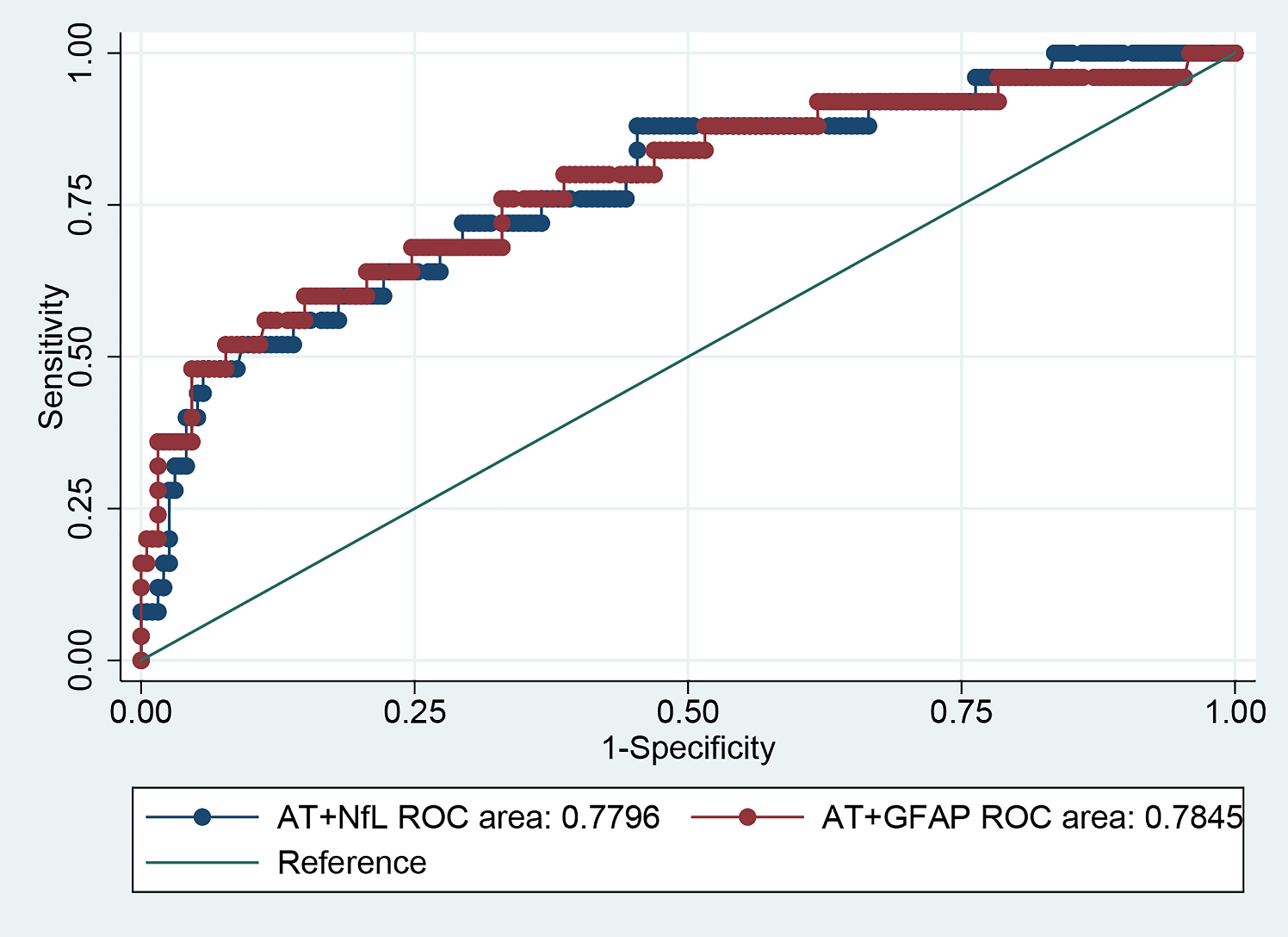


**Fig S8** The ROC curve and AUC of the optimal model in the validation cohort. Abbreviations: AUC, area under the ROC curve; ROC, receiver operating characteristic.


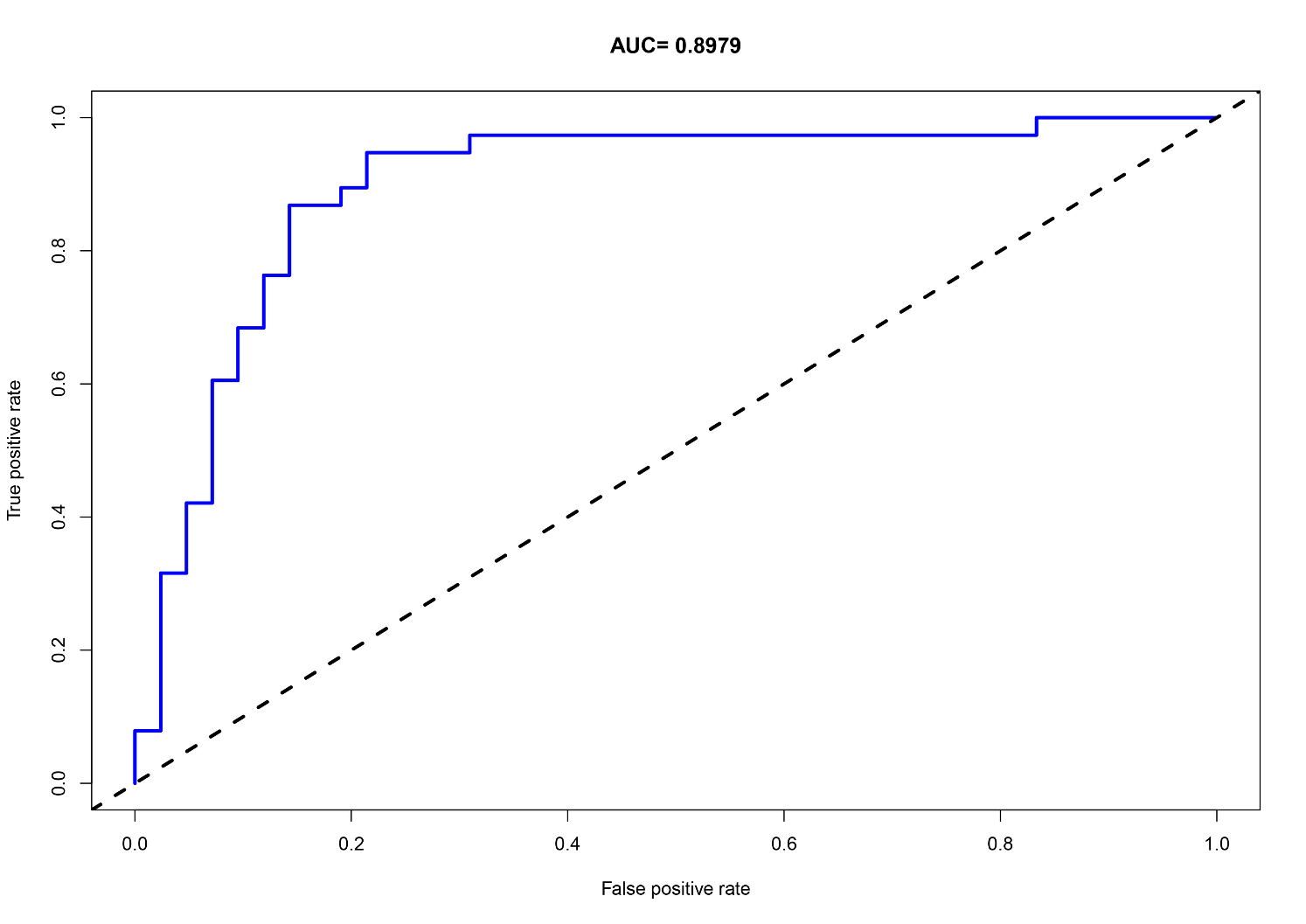


**Fig S9** Decision curve analysis on the optimal model using data from the ADNI cohort


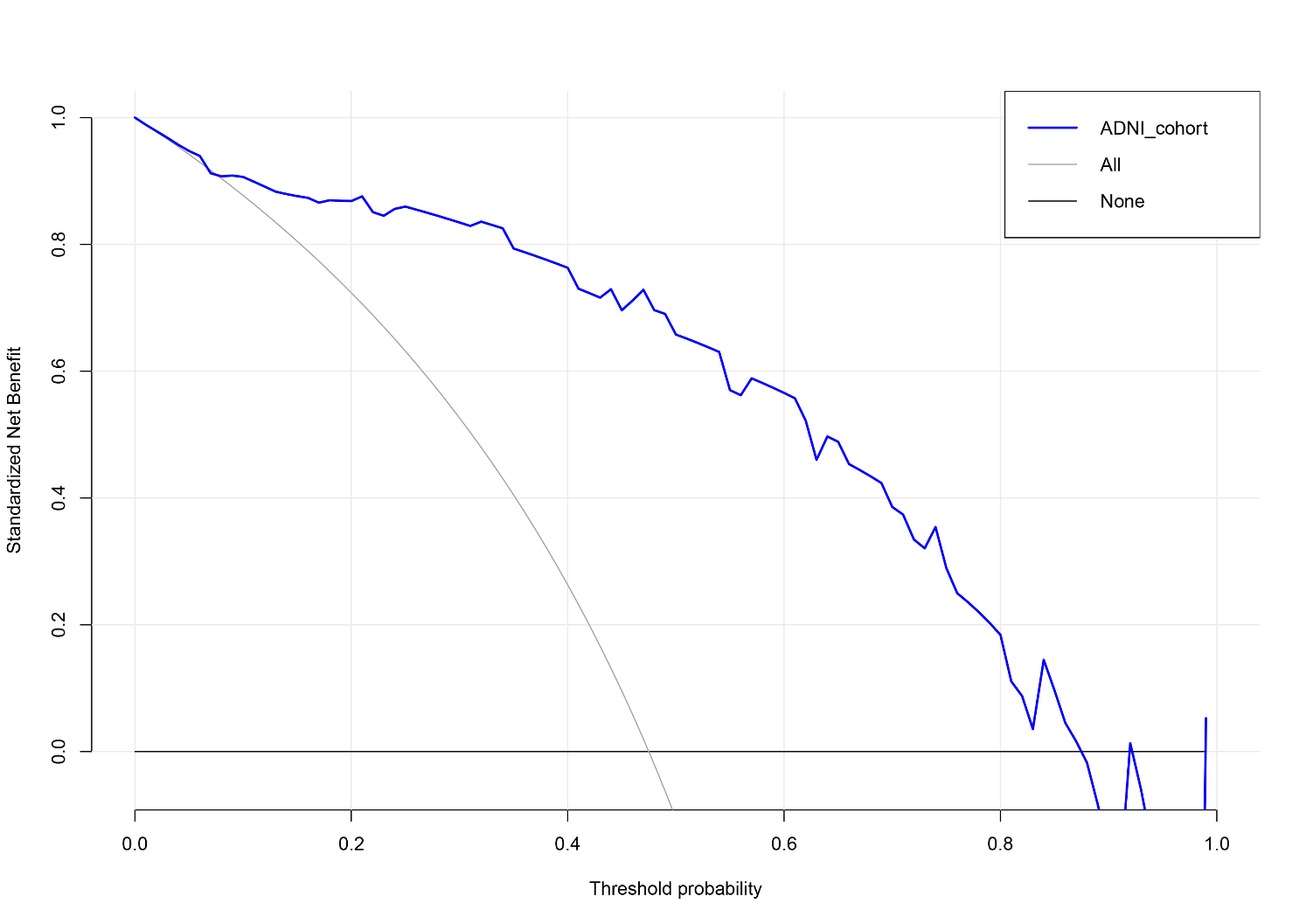


**Fig S10** Rad mediated the correlation between plasma GFAP concentration and MMSE scores.


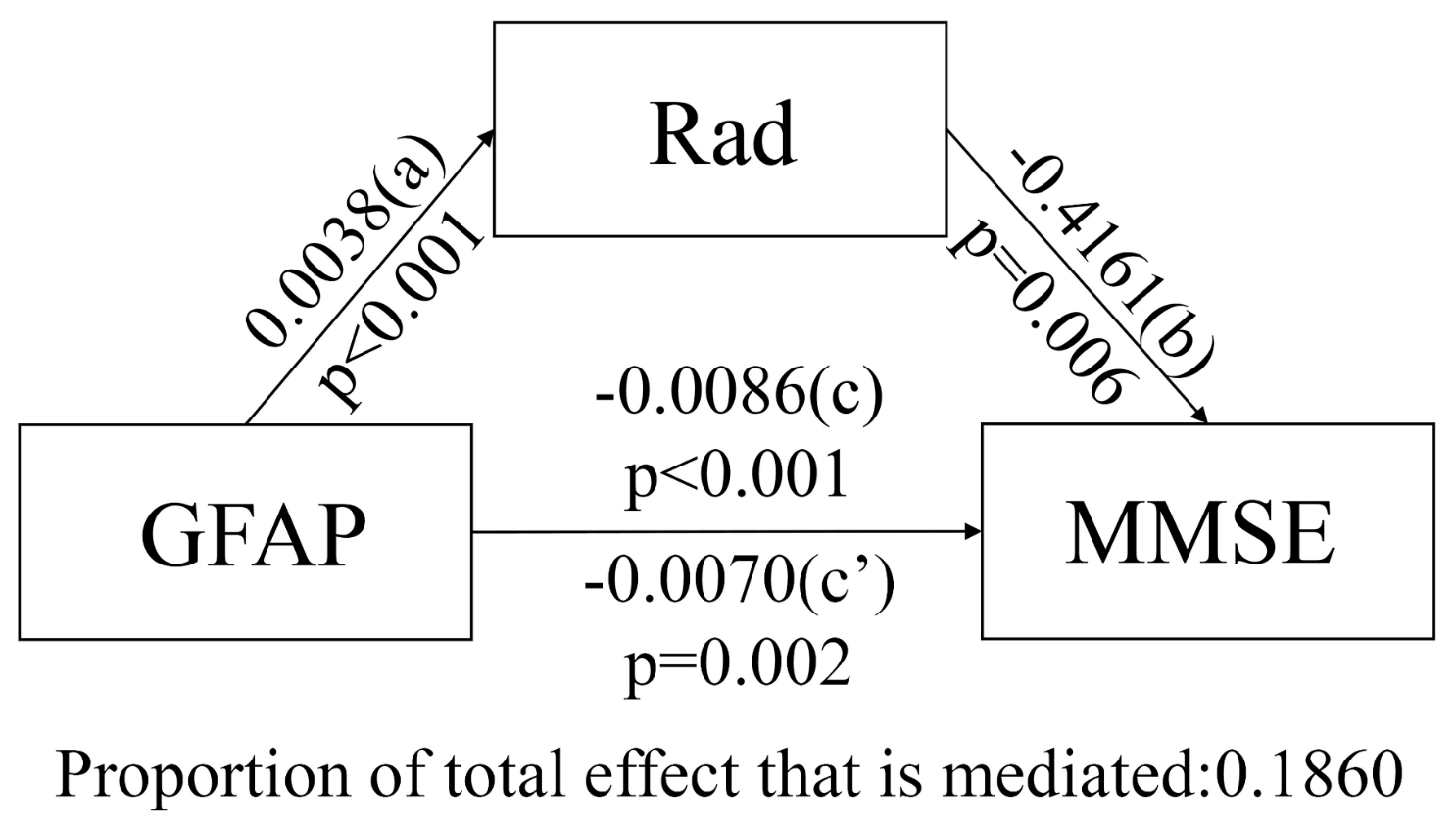


**Fig S11** Rad mediated the correlation between plasma NfL concentration and Outcome.


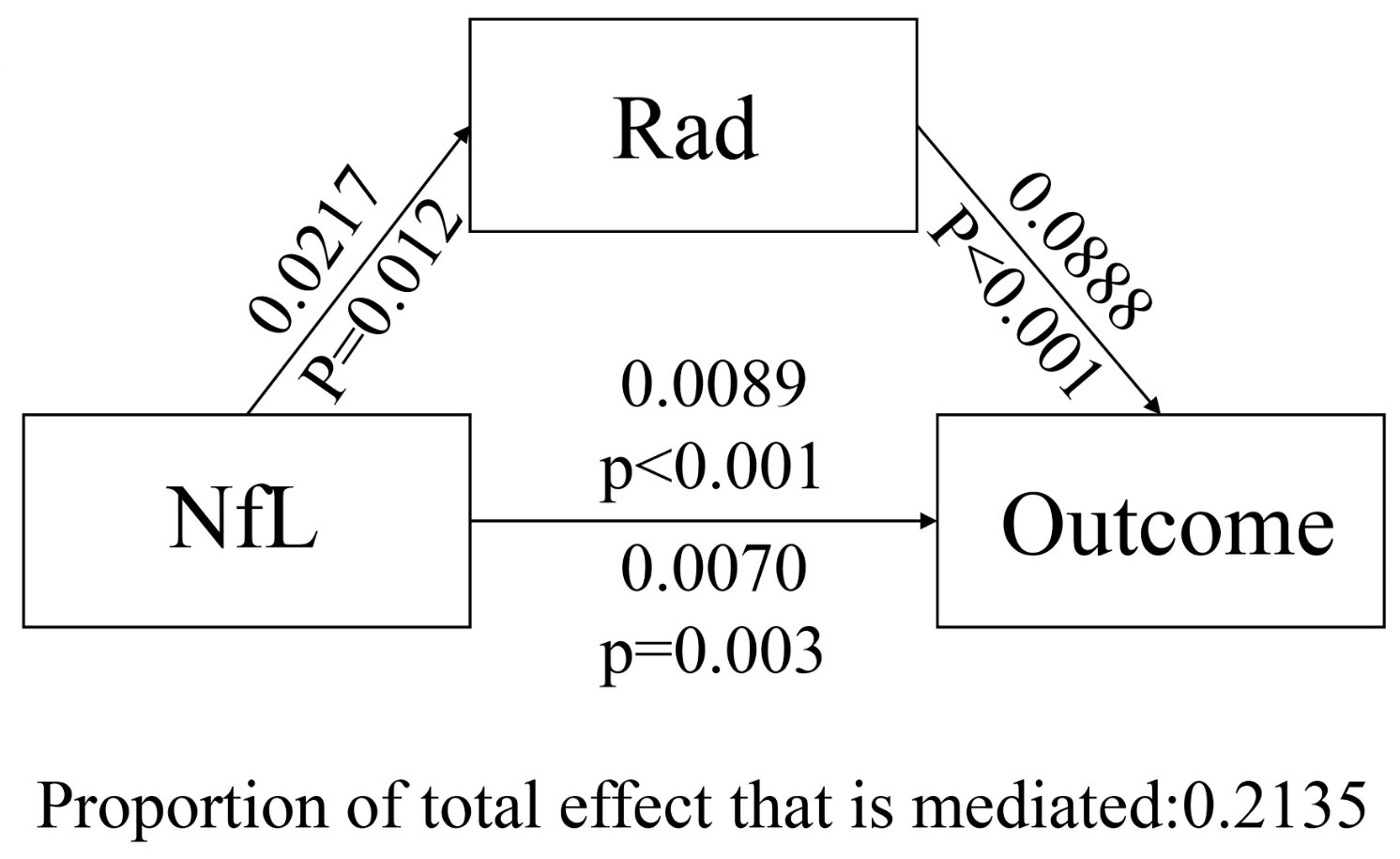


**
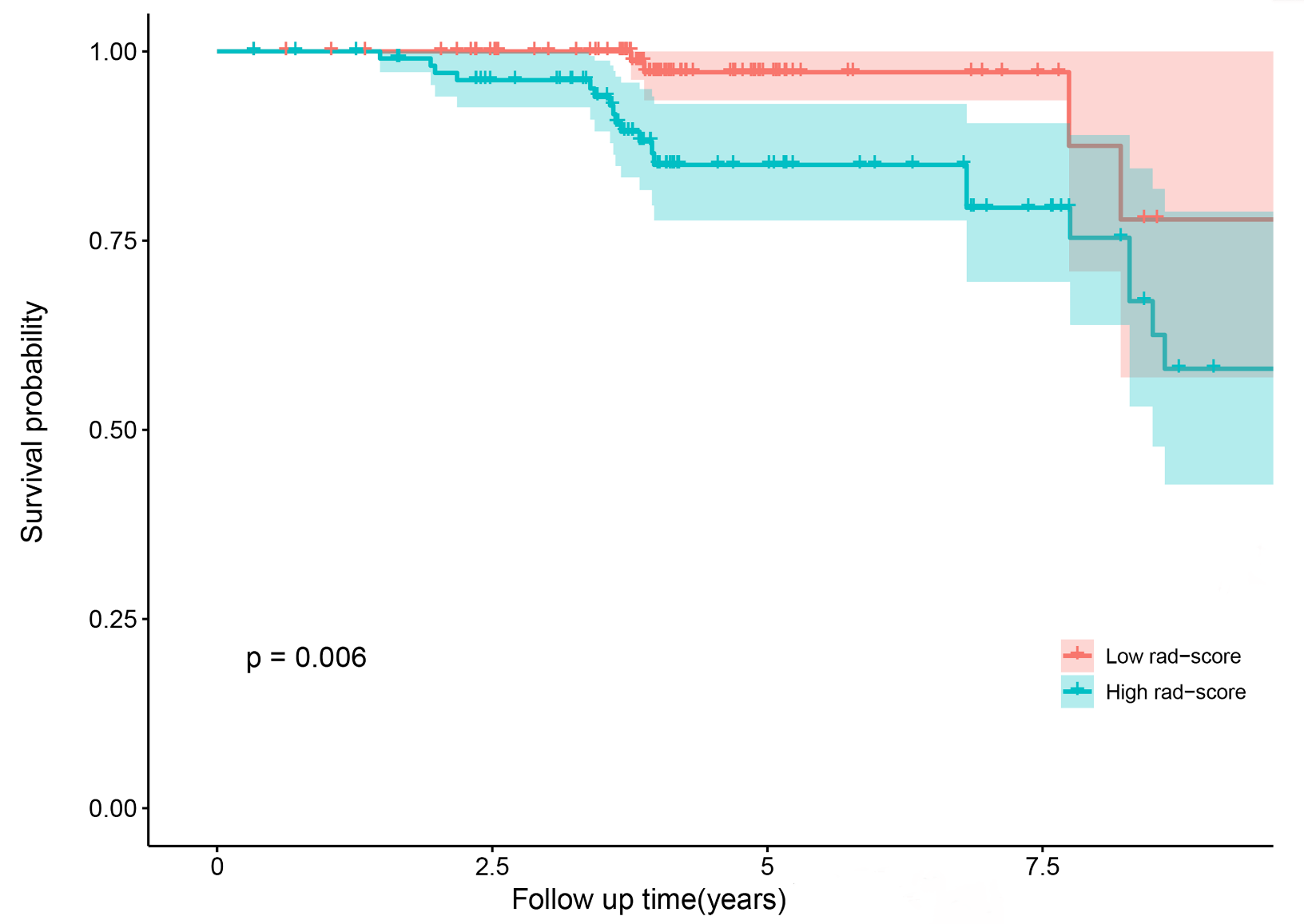
Fig S12** According to the Kaplan-Meier survival curve stratified by Rad scores.

**Reference**

[1]Tzourio-Mazoyer N, Landeau B, Papathanassiou D, et al. Automated anatomical labeling of activations in SPM using a macroscopic anatomical parcellation of the MNI MRI single-subject brain. Neuroimage 2002;15:273-89.

[2] Haralick, R. M., Shanmugam, K., & Dinstein, I. (1973). Textural features for image classification. IEEE Transactions on Systems, Man and Cybernetics, smc 3(6), 610-621.

[3] Assefa, D., Keller, H., M nard, C., Laperriere, N., Ferrari, R. J., & Yeung, I. (2010). Robust texture features for response monitoring of glioblastoma multiforme on T1 -weighted and T2 -FLAIR MR images: A preliminary investigation in terms of identification and segmentation. Medical Physics, 37(4), 1722-1736.

[4] Thibault, G. (2009). Indices de formes et de textures: de la 2D vers la 3D. Application au classement de noyaux de cellules. PhD Thesis, Universit AIX-Marseille: p.172.

[5] Aerts, H.J.W.L. et al. Decoding tumour phenotype by noninvasive imaging using a quantitative radiomics approach. Nat. Commun. 5:4006 doi: 10.1038/ncomms5006 (2014).

[6] Galloway, M. M. (1975). Texture analysis using gray level run lengths. Computer Graphics and Image Processing, 4(2), 172-179.

[7] Chu, A., Sehgal, C. M., & Greenleaf, J. F. (1990). Use of gray value distribution of run lengths for texture analysis. Pattern Recognition Letters, 11(6), 415-419.

[8] Dasarathy, B. V., & Holder, E. B. (1991). Image characterizations based on joint gray level-run length distributions. Pattern Recognition Letters, 12(8), 497-502.

[9] Thibault, G., Fertil, B., Navarro, C., Pereira, S., Cau, P., Levy, N., Mari, J.-L. (2009). Texture Indexes and Gray Level Size Zone Matrix. Application to Cell Nuclei Classification. In Pattern Recognition and Information Processing (PRIP) (pp. 140-145).

[10] Galloway, M. M. (1975). Texture analysis using gray level run lengths. Computer Graphics and Image Processing, 4(2), 172-179.

[11] Chu, A., Sehgal, C. M., & Greenleaf, J. F. (1990). Use of gray value distribution of run lengths for texture analysis. Pattern Recognition Letters, 11(6), 415-419.

[12] Dasarathy, B. V., & Holder, E. B. (1991). Image characterizations based on joint gray level-run length distributions. Pattern Recognition Letters, 12(8), 497-502.

[13] Thibault, G., Fertil, B., Navarro, C., Pereira, S., Cau, P., Levy, N., Mari, J.-L. (2009). Texture Indexes and Gray Level Size Zone Matrix. Application to Cell Nuclei Classification. In Pattern Recognition and Information Processing (PRIP) (pp. 140-145).

[14] Amadasun, M., & King, R. (1989). Textural Features Corresponding to Textural Properties. IEEE Transactions on Systems Man and Cybernetics,19(5), 1264-1274.
