# Supplementary figures and images for: Innovative Multivariable Model Combining MRI Radiomics and Plasma Indexes Predicts Alzheimer’s Disease Conversion: Evidence from a 2-Cohort Longitudinal Study
Source: Research (Wash D C). 2024 Apr 16;7:0354. doi: 10.34133/research.0354 (PMC11070845; doi:10.34133/research.0354)

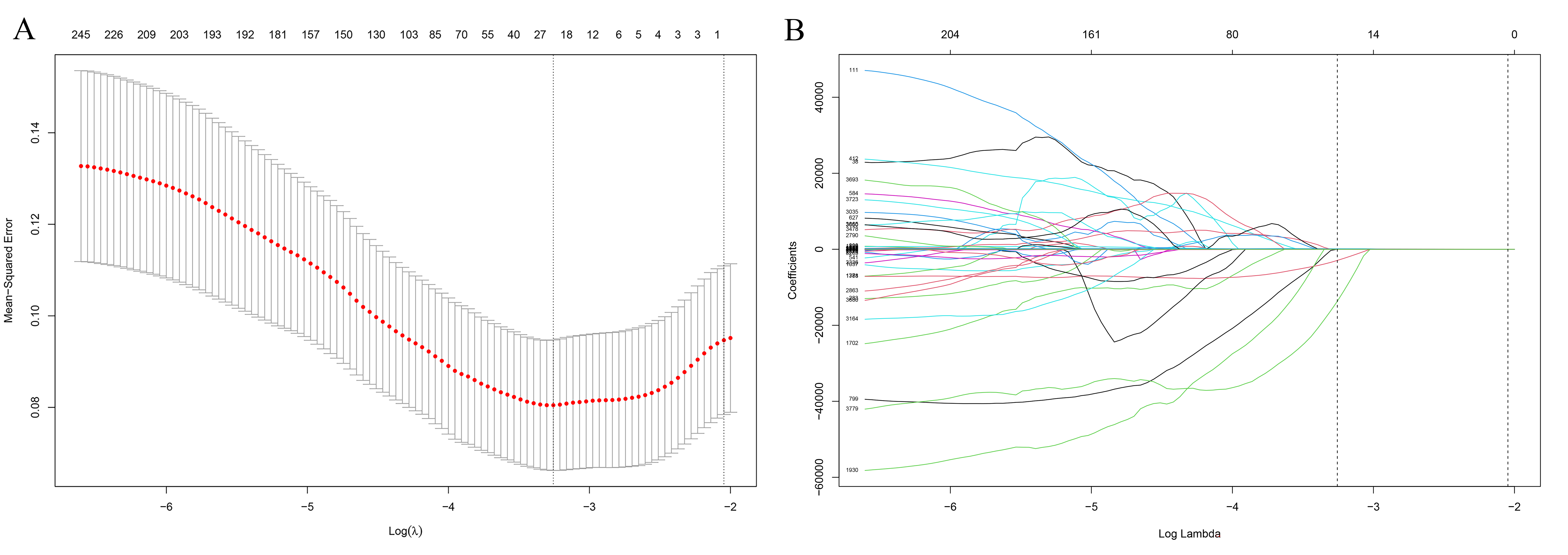

Supplement: Supplementary 1 — Figs. S1 to S12 [file research.0354.f1.zip › FigS1.tif]

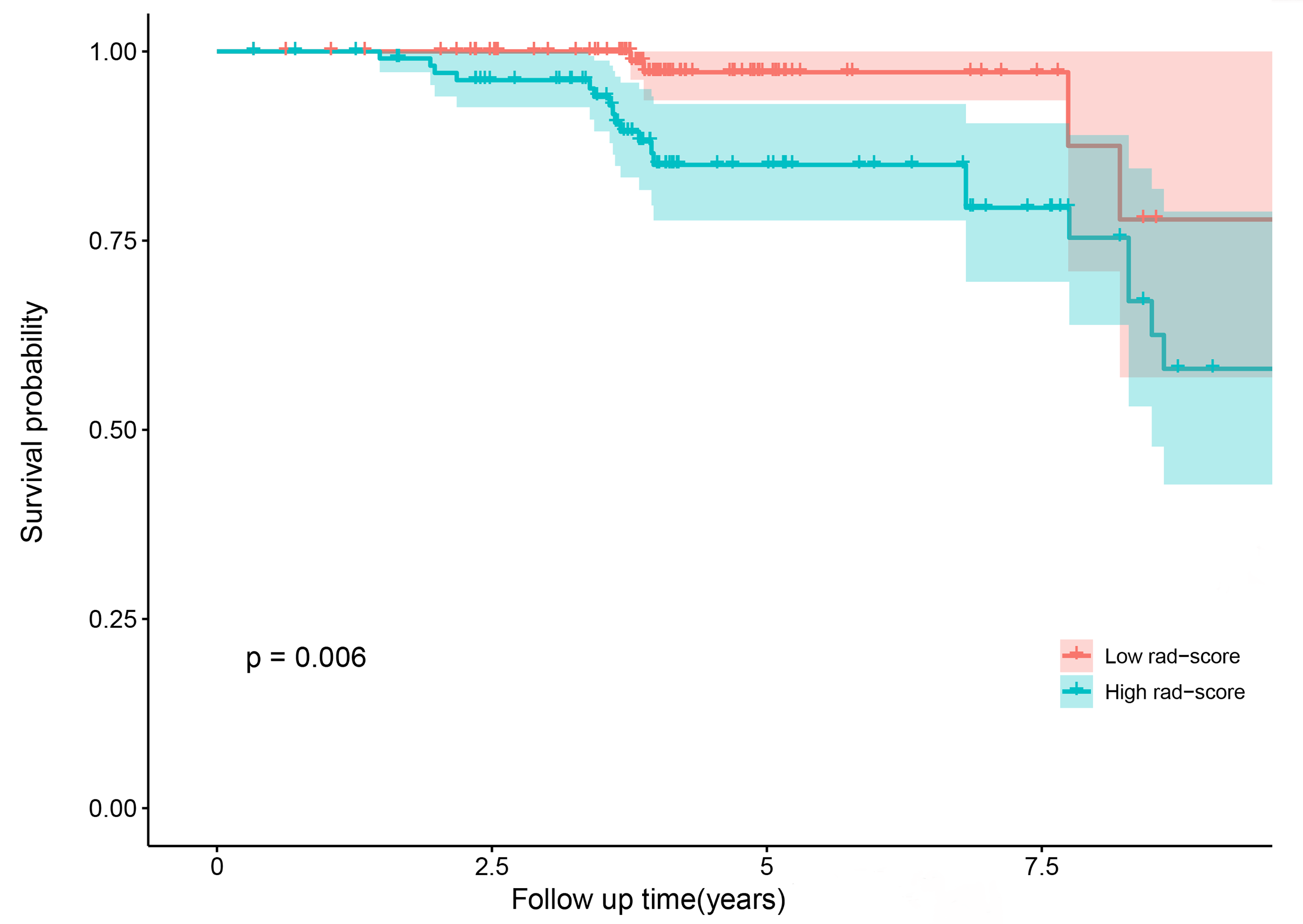

Supplement: Supplementary 1 — Figs. S1 to S12 [file research.0354.f1.zip › FigS11.tif]

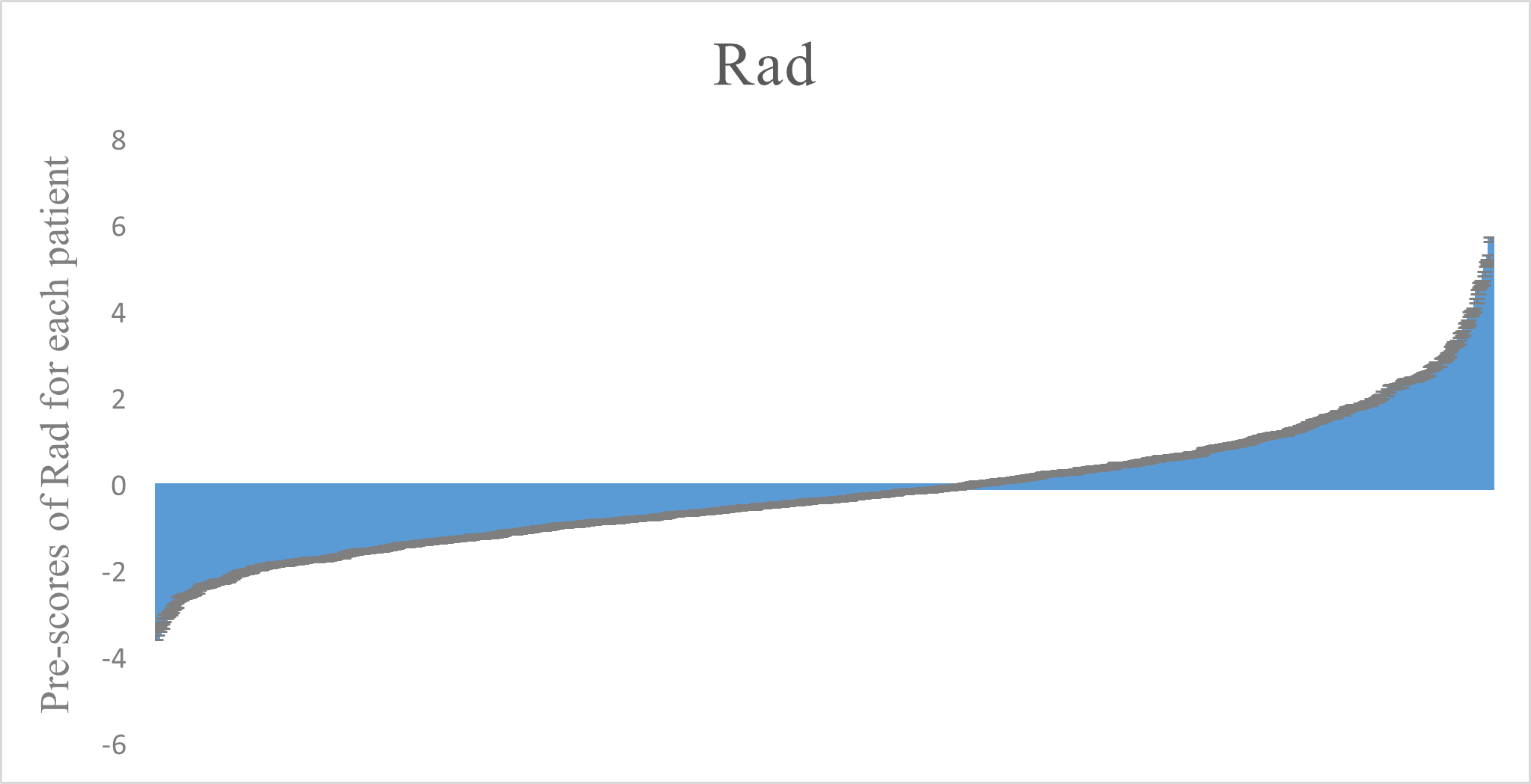

Supplement: Supplementary 1 — Figs. S1 to S12 [file research.0354.f1.zip › FigS2.tif]

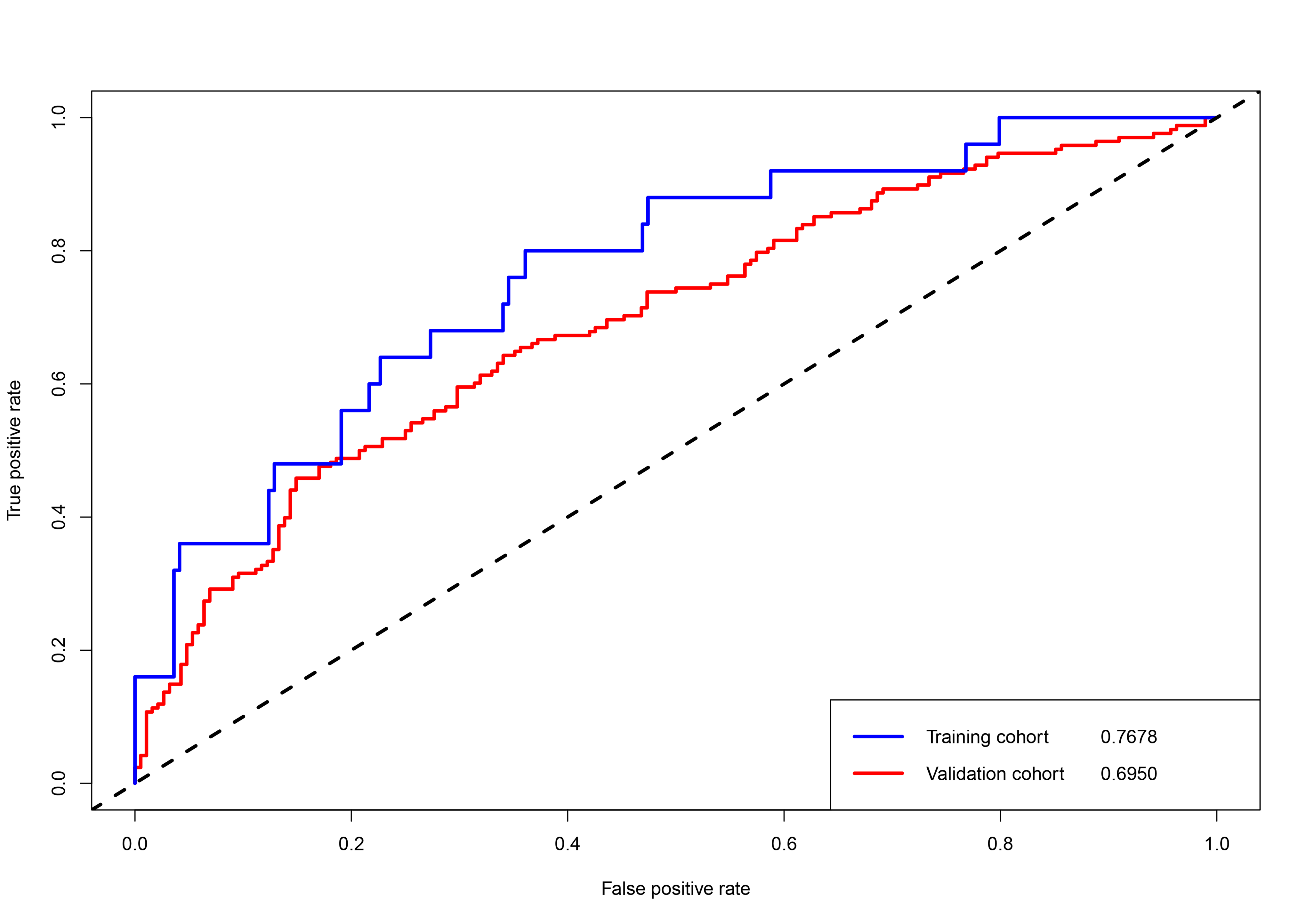

Supplement: Supplementary 1 — Figs. S1 to S12 [file research.0354.f1.zip › FigS4.tif]

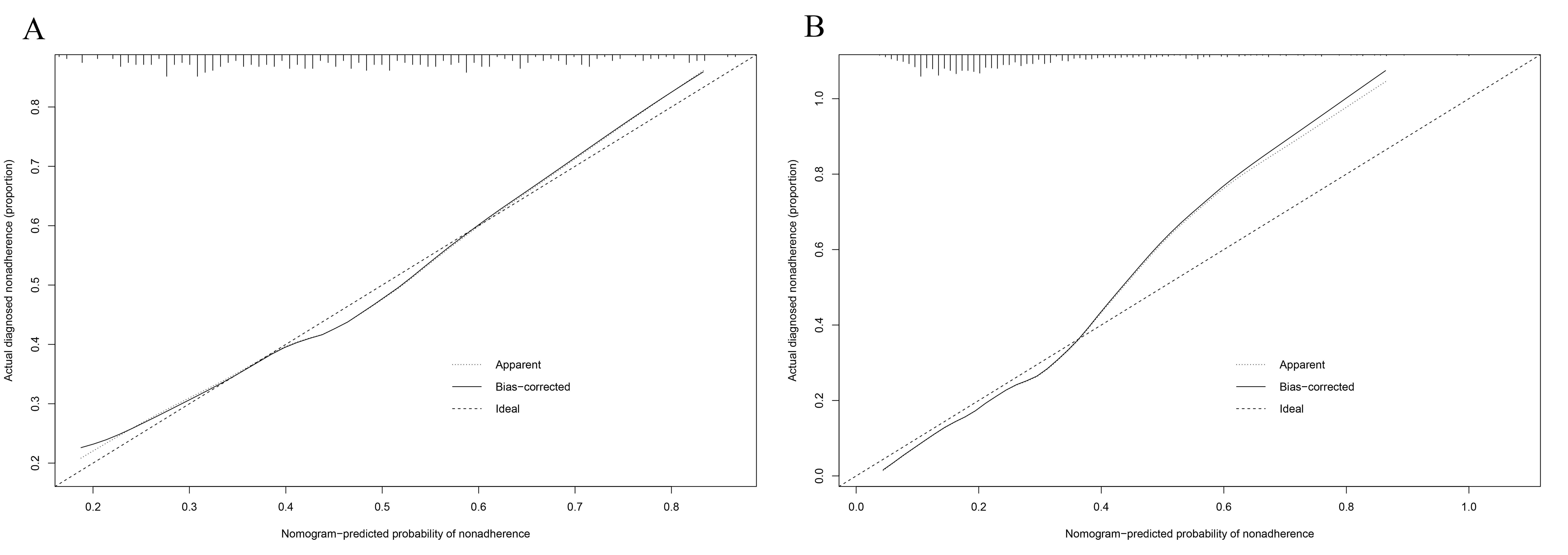

Supplement: Supplementary 1 — Figs. S1 to S12 [file research.0354.f1.zip › FigS5.tif]

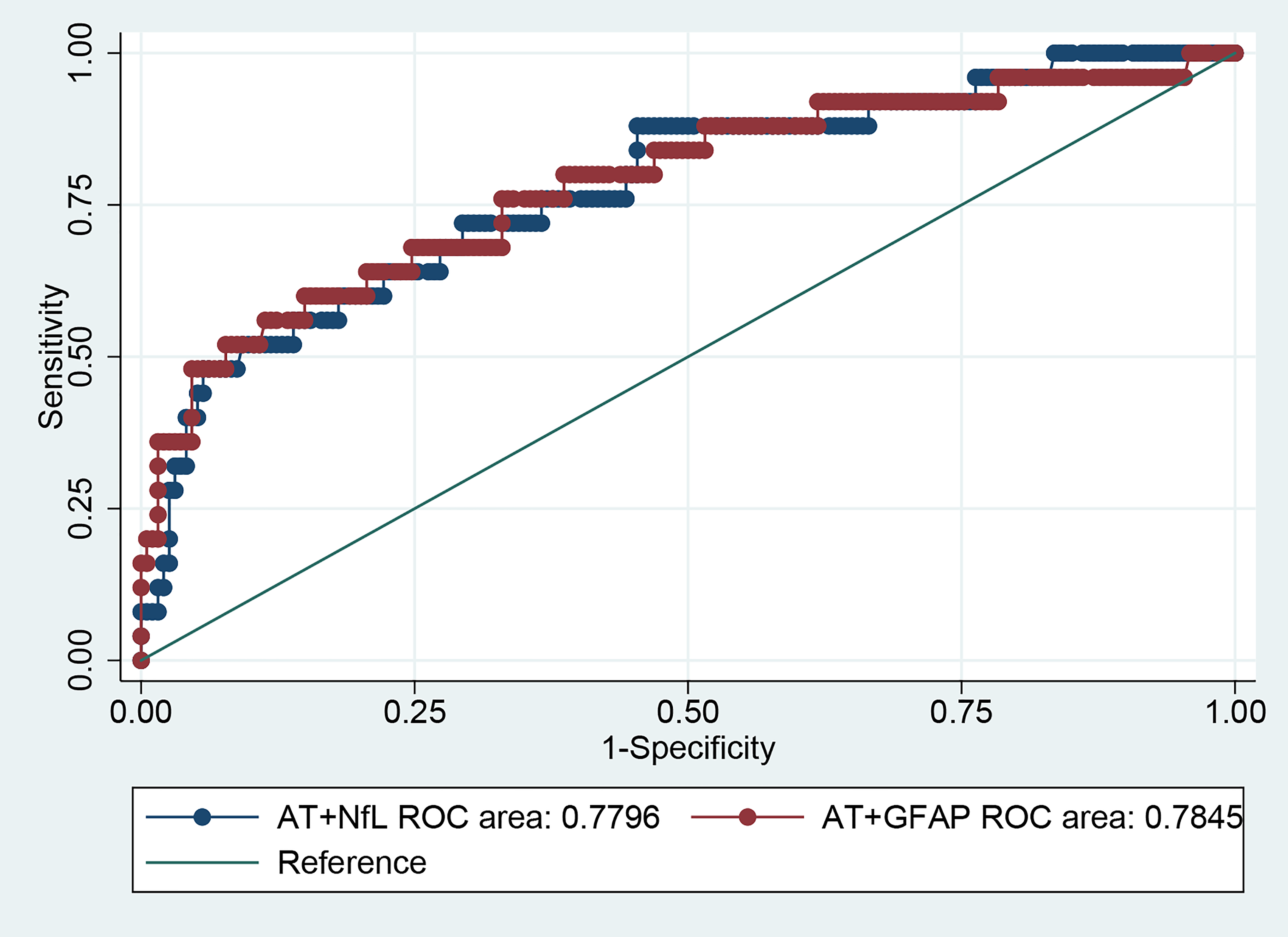

Supplement: Supplementary 1 — Figs. S1 to S12 [file research.0354.f1.zip › FigS6.tif]
